# Allelic Richness following Population Founding Events – A Stochastic Modeling Framework Incorporating Gene Flow and Genetic Drift

**DOI:** 10.1371/journal.pone.0115203

**Published:** 2014-12-19

**Authors:** Gili Greenbaum, Alan R. Templeton, Yair Zarmi, Shirli Bar-David

**Affiliations:** 1 Department of Solar Energy and Environmental Physics, The Jacob Blaustein Institutes for Desert Research, Ben-Gurion University of the Negev, Midreshet Ben-Gurion, Israel; 2 Mitrani Department of Desert Ecology, The Jacob Blaustein Institutes for Desert Research, Ben-Gurion University of the Negev, Midreshet Ben-Gurion, Israel; 3 Department of Biology, Washington University, St. Louis, Missouri, United States of America; 4 Institute of Evolution, and Department of Evolutionary and Environmental Biology, University of Haifa, Haifa, Israel; Tel Aviv University, Israel

## Abstract

Allelic richness (number of alleles) is a measure of genetic diversity indicative of a population's long-term potential for adaptability and persistence. It is used less commonly than heterozygosity as a genetic diversity measure, partially because it is more mathematically difficult to take into account the stochastic process of genetic drift for allelic richness. This paper presents a stochastic model for the allelic richness of a newly founded population experiencing genetic drift and gene flow. The model follows the dynamics of alleles lost during the founder event and simulates the effect of gene flow on maintenance and recovery of allelic richness. The probability of an allele's presence in the population was identified as the relevant statistical property for a meaningful interpretation of allelic richness. A method is discussed that combines the probability of allele presence with a population's allele frequency spectrum to provide predictions for allele recovery. The model's analysis provides insights into the dynamics of allelic richness following a founder event, taking into account gene flow and the allele frequency spectrum. Furthermore, the model indicates that the “One Migrant per Generation” rule, a commonly used conservation guideline related to heterozygosity, may be inadequate for addressing preservation of diversity at the allelic level. This highlights the importance of distinguishing between heterozygosity and allelic richness as measures of genetic diversity, since focusing merely on the preservation of heterozygosity might not be enough to adequately preserve allelic richness, which is crucial for species persistence and evolution.

## Introduction

Genetic diversity is an important aspect of the dynamics of populations, as it is directly related to the evolutionary potential of the population and the deleterious effects of inbreeding [1]. There are, however, several different types of measures of genetic diversity, most notably measures based on heterozygosity and measures based on allelic richness (defined as the number of alleles). These groups of measures differ in their formulations, their ecological and evolutionary interpretations, and the mathematical frameworks in which they can be applied [2]–[4].

Observed heterozygosity (*H_O_*), the frequency of heterozygous individuals in a population, or expected heterozygosity (*H_E_*), the probability that two gametes, randomly chosen from the gene pool, are of different alleles, are by far the measures most commonly used by the majority of papers that present a genetic summary of populations [4] (e.g., [5], [6]). These measures are very sensitive to the allele frequencies in the population, rather than just to the number of alleles. It has been shown that a decrease in the observed heterozygosity can induce a decrease in the average fitness of individuals [7], [8], and thus this measure has clear ecological consequences. There exists an extensive mathematical framework, F-statistics, first presented by Wright [9], that allows for the modeling and analysis of varied scenarios regarding heterozygosity, as well as the inclusion of processes such as gene flow to provide quantitative predictions and assessments. Allele richness (also referred to as allelic diversity) is calculated as the average number of alleles per locus [1]. A decrease in the allelic richness could lead to a reduction in the population's potential to adapt to future environmental changes, since this diversity is the raw material for evolution by natural selection [10]. While not all variation is related to the adaptive potential, clearly no standing variation exists if no allelic richness exists. Moreover, there is evidence that high allelic richness, even of merely neutral alleles, increases evolvability by making a larger fraction of the genotypic space accessible by fewer mutational events [11]. Allelic richness is, therefore, a strong indicator for the evolutionary potential of a population [2], [3], [12], and it has been suggested that this measure is of key importance in population conservation and management [13]–[18]. Allelic richness measures are also commonly presented in population genetic summaries (as the number of alleles in a given locus or the mean number of alleles per locus). However, in practice, conclusions pertaining to these measures are often merely comparative, such as “population *A* has higher allelic richness than population *B*” or “the population had higher allelic richness at time *T* than at time *S*”, and not quantitative.

As human activity pushes many species closer to extinction, conservation of populations is increasingly becoming a major concern, and an important aspect of conservation is addressing the preservation of genetic diversity [19]. Conservation programs combine general rules and guidelines, usually derived from the F-Statistics framework, with the specific ecological scenario in question [12], [20]. One such general rule, concerning gene flow, is the “One Migrant per Generation” (OMPG) rule [12], [21]. The rule states that, under the conditions of an island model of migration in which gene flow is not influenced by distance or any geographical feature, an exchange of one effective migrant per generation (effective migrants are migrants that breed and contribute to the population's gene pool; for the purpose of this paper, all migrants will be regarded as effective migrants) is enough to adequately maintain the genetic diversity of the population [22], [23]. This rule is derived from the island model [24], and other F-statistics models have been formulated (e.g., the stepping stone model [25]), but they are used less frequently as conservation guidelines.

Founder events are known to decrease the genetic diversity of the population [26], and are often followed by a demographic expansion. It has been shown, both theoretically [2], [26]–[30] and empirically [27], [31], that allelic richness is more sensitive than heterozygosity to founder events followed by expansions, since allelic richness does not consider abundances of the alleles but only their presence (a rare allele that is lost in a founder event will probably not affect heterozygosity much, but the loss does reduce allelic richness). Moreover, allelic richness is more indicative of the evolutionary potential of the population in the long-run in these scenarios [2], [3], [12], as the existence of alleles, rather than their frequencies, holds a significant part of potential for response to selection, as selection limits are determined by the initial allelic composition [13], [32] (studied for biallelic loci by [33]–[35]) rather than by levels of heterozygosity. For example, consider a population with *n* different alleles with different selection coefficients (e.g., taken randomly from a uniform distribution *U(0,1)*). If we consider that only selection is at play, eventually alleles with the highest selection coefficients will become abundant while the rest will be lost. Thus the eventual fitness of the population will be determined by the expected maximal value of the initial selection coefficients, a value which depends on *n*, the number of alleles (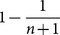 in the case of the uniform distribution above). This maximal value is higher when *n* is larger, and therefore, populations with higher allelic richness are expected to eventually show higher average fitness, under these assumptions. Nevertheless, most theoretical models and conservation applications pertaining to these scenarios are still drawn from the F-Statistics framework [4], where higher heterozygosity levels are considered to convey increased response to selection. While some work has been done in modeling allelic richness [36]–[39], the field has not yet been fully investigated.

Founder events are often followed by a loss of alleles, referred to as the “founder effect” [40]. The founders of the new population seldom carry all the alleles that existed in the original population. This loss of alleles might later be countered by gene flow induced by migrants arriving from the source population carrying the lost alleles, and therefore, gene flow is a force that may recover allelic richness, as well as heterozygosity [41]. Since founded populations are usually small, genetic drift – the stochastic element of the genetic process that can lead to the loss of alleles – is another relevant genetic force that needs to be taken into account. These two forces can be seen as acting in opposing directions in the founded population, with gene flow potentially generating an increase in allelic richness and genetic drift leading to a decrease.

The goal of this paper is to present a simulation framework, to be used as a method for estimating the maintenance and recovery of allelic richness, as well as for identifying the appropriate statistical properties with which to address allelic richness in similar modeling frameworks. A model was developed in a neutral theory [42], [43] context that focuses on gene flow and genetic drift in a founder scenario. The model tracks and simulates a single allele lost in the founder event under different demographic parameters and allele frequencies, and later it is shown how the single-allele results can be extended and applied to the entire allele frequency spectrum to give a meaningful estimation of overall allelic richness recovery. While the model does not include mutation and selection, although they may be relevant in many scenarios, it provides insights into the potential effect of gene flow in the recovery of allelic richness. The model's results also demonstrate that the OMPG rule may not be sufficient to conserve allelic richness, and thus they emphasize the need to distinguish between allelic richness and heterozygosity regarding management conclusions pertaining to migration rates between populations. Such conclusions should be drawn while taking both measures into account.

## Methods

### 2.1. Model

The model consists of a source population and a newly founded population. The source population is the population from which the founded population originated and from which migrants can possibly arrive. It is assumed to be an ideal population at Hardy-Weinberg equilibrium, much larger than the founded population, and is therefore assigned a static genetic description (i.e., allele frequencies in the population do not change over time), while the founded population is dynamic in this regard. The model tracks the frequency of a single allele in the founded population over time. The founded population is assumed to be demographically expanding with a discrete logistic equation describing the population size:
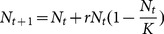
(1)where *N_t_* is the population size at time *t*, *r* is the population growth rate and *K* the carrying capacity (note that migration does not affect the population size). *N_t_* was rounded to the nearest integer to delineate the population size for the purpose of performing simulations. Since the goal of the model is to assess the potential of migration to negate allele loss as a result of the founder effect, the allele in question is assumed to have been lost during the founding process, and therefore, its initial frequency is 0. The same allele has a constant frequency of *Q* in the source population.

The model assumes a Poisson-distributed migration pattern. The mean number of migrants per generation from the source population to the founded population is *M* and the mean number of migrants from the founded population back to the source population is *mN_t_* (a proportion *m* of the population each generation). Migration is defined to be asymmetric because we assume the source population is large and static, while the size of the founded population is dynamic (following eq. 1). Note that the inclusion of back-migration when the source population is constant could have been left out of the model, since these migrating individuals are effectively “lost”, but it has been included in order to provide an outline for future development of the model (here we assume that the source population is very large, and therefore, the effect of back migration on the source population allele frequencies is negligible). We also used a deterministic migration pattern, in which the number of migrants per generation is constant (see S1 Appendix and S1 and S2 Tables). Migrants are assumed to carry the allele in question according to the frequency of the allele in the population from which they are migrating.

Genetic drift is simulated according to the Wright-Fisher model [24]: a random independent draw for each gamete determines whether it is a gamete of the allele in question or not. This induces a binomial distribution on the number of gametes of the allele. The model, combining both gene flow and genetic drift, can thus be summarized as follows:
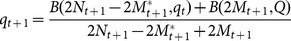
(2)with the initial condition 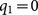. Here *q_t_* is the allele frequency; *B(n,p)* is the Binomial distribution with *n* number of trials and success probability *p*; 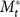, the number of migrants back-migrating to the source population at time *t*, is a random variable with a Poisson distribution 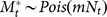; and *M_t_*, the number of migrants to the founded population at time *t*, is a random variable with a Poisson distribution 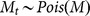. The numerator represents the number of alleles in the population after one generation of migration as the sum of two random variables with the first reflecting alleles drawn from the founded population's gene pool and the second alleles drawn from the migrants, and the denominator represents the relevant population size.

The expected value of *q_t_* is thus given by: 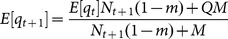
(3)and at equilibrium, the expected value is 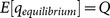 (for 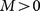). This theoretical expected value should be approached when the stochastic nature of the process (i.e. genetic drift) is negligible, but otherwise, the empirical mean allele frequency at equilibrium, 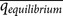, might also depend on other parameters, due to the absorbing boundaries of the genetic drift process at 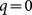 and 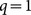.

A comprehensive theory for analytically describing 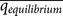 that takes all the model parameters into account is not yet available, but a simplified approximation can be used. The expected waiting time between arrival from the source population of two individual alleles is 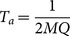 as 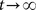, and the expected waiting time for an individual allele's loss, assuming that the population is at carrying capacity (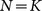), is approximately 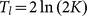 [44] (here an additional assumption is made, that the allele frequency at arrival is very small compared to *K*). Thus, for 
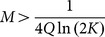
, the waiting time between arrivals is shorter than the loss time (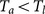), and we expect 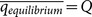 to hold. Otherwise, 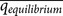 is expected to be lower in proportion to the time interval in which the allele is present:
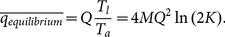
(4)


### 2.2. Simulations

Simulations were carried out for different scenarios with the following parameter values: initial population size 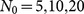; carrying capacity 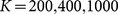; population growth rate 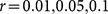. All scenarios were simulated for frequencies in the source population of 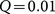 to 0.02 (0.01 intervals) and migration rates from the source population to the founded population of 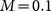 to 30 (0.1 intervals). For all simulations, a fixed back-migration value of 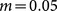 was used. These parameters were chosen to illustrate the presented framework since they could be reasonable parameters for a reintroduction of a mammalian population, as a possible application, but the framework is not restricted to these parameters.

We ran 1000 simulations for each scenario. The mean allele frequency and the probability of allele presence (see below) were calculated for each generation across the 1000 simulations. Each of these simulations was run until equilibrium was reached, plus an additional 200 generations to generate the equilibrium phase of the system (equivalent to the migration-drift balance). The equilibrium generation was defined by first defining an equilibrium separately for the mean allele frequency and for the probability of allele presence. Equilibrium was defined as the generation for which the following 100 generations show no trend, i.e., the difference between the number of generations showing a positive change in the statistical property (mean allele frequency or probability of allele presence) and the number of generations showing a negative change was one or zero. The overall equilibrium was taken as the greater of these two equilibria to ensure that the system is stable for both statistical properties. This conservative definition of equilibrium, which could overestimate the actual time needed to reach equilibrium, was chosen to ensure that the analysis is done within the equilibrium phase rather than to determine precisely the time needed to reach equilibrium.

The model focuses on time scales that are sufficiently short and on population sizes that are sufficiently small, so that mutation can be neglected; mutation events are very rare in small populations during short time scales [20]. The model was simulated using MATHEMATICA software [45], and appears in S2 Appendix along with the code for the analysis and the graphical representation of the results (which could be used with alternative parameterizations).

### 2.3. Simulation analysis

**Figure 1:**
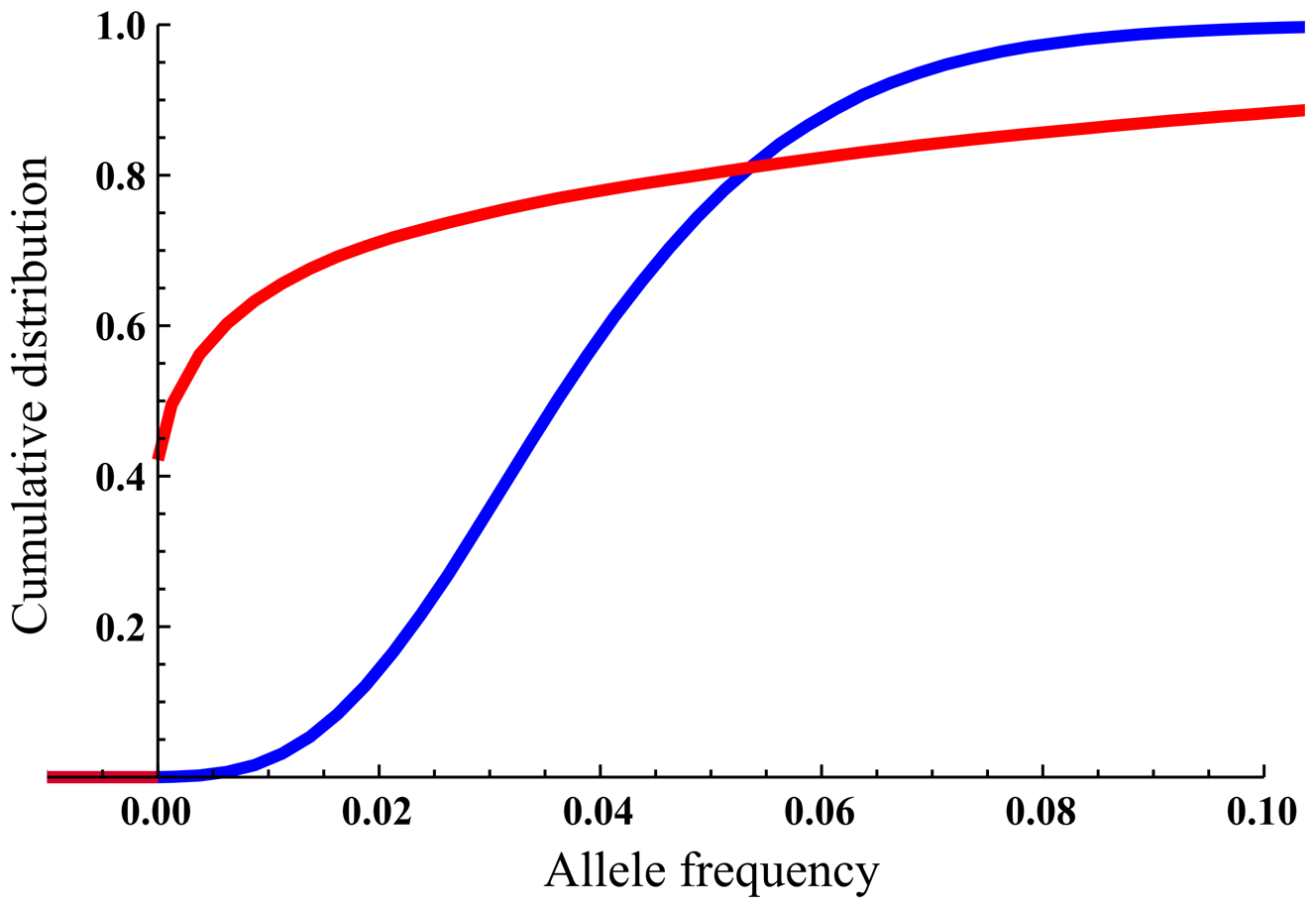
Cumulative density functions for the allele frequency at the equilibrium phase. 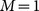
 migrants in blue, 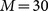 migrants in red; scenario parameters 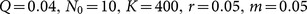. The jump at allele frequency 0 for 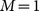 is due to the low 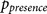 for this scenario.

Probability measures can be broken into a discrete part and a continuous part (Jordan's decomposition theorem [46]). In the dynamics of allele frequency over time, the discrete part of the probability measure constitutes the allele frequencies 0 and 1 - the events of allele fixation and allele loss, respectively. For each of these two points, there is a positive probability that the allele has those frequencies, and we define these probabilities as 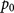 and 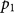 respectively. This attribution of positive probabilities to singular points is due to the fact that the stochastic process of genetic drift has absorbing boundaries at these points, and alleles that have reached loss or fixation are expected to remain in these states for some time (permanently in the absence of gene flow or mutations). For the frequencies between but not including 0 and 1, a continuous probability distribution is an appropriate description. Thus, the behavior of the system at 0 and 1 should be considered separately from the rest of the distribution (see Fig. 1 for an illustration, explained below). We define the ‘probability of allele presence' as the probability of the allele not being at frequency zero:
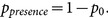
(5)


The probability 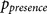 was calculated for each generation in each scenario from the simulations.

The equilibrium phase of the population was defined as the 200 generations after equilibrium was reached, and the mean 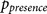 was calculated over these 200 generations, as well as the mean allele frequency (the mean over 200 generations of the mean allele frequency for 1000 simulations). For a given scenario, the minimal migration rate required to reach 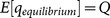 (i.e., the mean allele frequency at equilibrium as the source population's allele frequency) was defined as the minimal migration rate 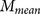 for which all 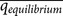 values for 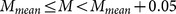 are above 0.95*Q* (i.e., the minimal migration for which five consecutive data points are above 95% of the allele frequency in the source population). This was done in order to estimate how many migrants are needed to ensure that the expected mean allele frequency at equilibrium is reasonably stable around *Q*.

The presence of an allele in the system was defined based on a threshold - the ‘95% probability of allele presence’. Only scenarios in which 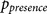 is higher than 95% (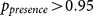) are assumed to have the allele in question with high enough confidence, while those below 95% are assumed to be missing the allele (used similarly by Tracy *et al.* [47]). For a given scenario, the migration rate required for allele presence with 95% probability, 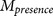, was defined as the minimal migration rate for which 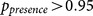. Both 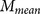 and 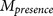 were obtained for all scenarios and for *Q* values of 0.02, 0.04, 0.1 and 0.2.

### 2.4. Source population allele frequency spectrum

In order to assess the model's implications for a polymorphic locus and not just a single allele, the allele frequency spectrum of the source population is compared with results for simulations with different *Q* values. To illustrate this process, and the application of the model with allele frequency spectra, examples of theoretical frequency spectra for the source population were generated using the method presented by Ewens [48]:
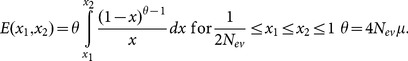
(6)


**Figure 2:**
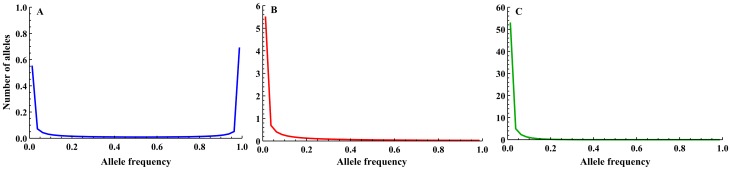
Theoretical allele frequency spectra. Theoretical allele frequency spectra for A) 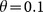 (blue); B) 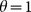 (red); C) 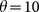 (green). The frequency spectra were generated using equation 6.

This equation allows for the calculation of the expected number of alleles with frequencies between 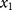 and 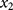, where *μ* is the mutation rate and *N_ev_* the variance effective population size of the source population. 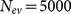 (although the population is assumed large, a finite *N_ev_* was used in order to derive an equilibrium allele frequency spectrum) and three different mutation rates, 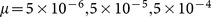 (resulting in 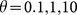, respectively), were taken to represent the source population. These per locus mutation rates correspond to the estimated range of human mutation rates [49], [50]. The resulting frequency spectra appear in Fig. 2.

The ‘95% probability of allele presence’, as described above, was similarly used to obtain a ‘cut-off frequency’, *Q_c_*, a threshold frequency (in the source population) above which the allele in question is assumed to be present in the population (i.e., 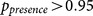). The cut-off frequency of a given scenario was then compared with the allele frequency spectra in Fig. 2, and the number of alleles above the cut-off frequency was calculated using eq. 6, i.e. 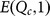, as well as their proportion from the overall expected number of alleles, i.e. 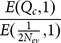.

The cut-off frequency and the number and proportion of alleles that are potentially expected to be recovered by gene flow (with the 95% probability guideline), if lost in a founder effect, were calculated for three different migration rates, 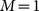, 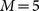 and 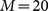.

## Results

**Figure 3:**
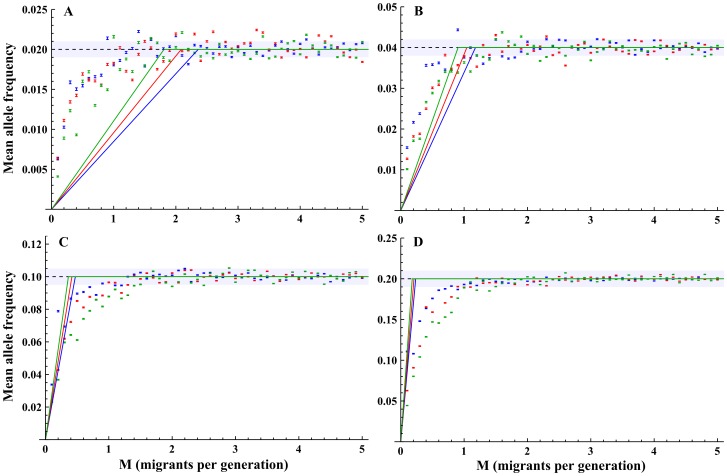
Mean allele frequencies at equilibrium (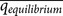) as a function of the number of migrants per generation. Solid lines indicate the estimation of the mean allele frequency (equation 4), dashed lines indicate 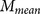 thresholds. 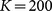 in blue; 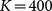 in red; 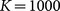 in blue. A) 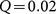; B) 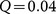; C) 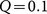; D) 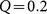. Scenario parameters: 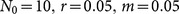. Error bars indicate the standard error of the mean.

In all simulated scenarios, the mean allele frequency in the founded population increased over time, showing eventual stabilization. The same pattern was observed for 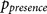. The number of migrants required for the mean allele frequency at equilibrium (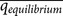) to recover to the same mean frequency as in the source population, 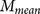, varied between 0.2 to 4.9 migrants per generation, with most scenarios requiring between 0.5–2.5 migrants, summarized in Table 1 (average over all scenarios simulated of 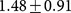 migrants). For migration rates below 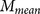, the mean frequency at equilibrium was lower than *Q*, with an example shown in Fig. 3 (all results are presented in S1–S9 Figs.). 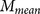 values were generally higher for rare alleles (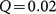 and 0.04), compared with more common alleles (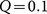 and 0.2), with averages of 1.74±0.98 migrants, and 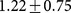 migrants respectively. 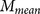 values were also generally higher for larger initial population sizes, higher carrying capacities and higher growth rates (Table 1).

**Table 1:** 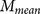
 and 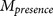 thresholds for different scenarios.

			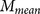				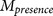			
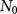	r	K	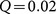	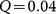	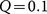	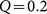	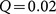	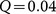	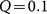	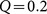
5	0.01	200	1.2	0.6	0.2	0.2	26.8	11.7	2.1	1.0
		400	0.7	1.4	0.4	0.2	30.4	5.9	1.9	0.8
		1000	0.8	0.4	0.6	0.2	16.6	10.8	2.0	0.7
	0.05	200	1.8	1.2	0.6	0.7	17.4	6.3	1.9	0.8
		400	2.2	0.4	0.5	1.0	12.5	5.0	1.6	0.8
		1000	1.9	0.8	0.7	0.8	9.1	3.8	1.3	0.6
	0.1	200	0.9	0.9	1.2	0.6	17.8	6.3	1.9	0.8
		400	1.4	1.7	1.4	1.2	12.4	4.9	1.6	0.8
		1000	1.3	2.5	1.3	1.5	9.3	3.9	1.4	0.7
10	0.01	200	1.8	1.1	0.7	1.3	19.8	7.7	2.5	0.9
		400	1.0	0.4	0.7	0.5	15.7	10.4	1.9	0.8
		1000	1.0	0.5	0.5	0.3	13.0	4.7	1.8	1.0
	0.05	200	1.6	1.9	1.3	1.0	17.3	6.4	1.9	0.8
		400	1.9	1.3	1.4	1.2	12.9	4.9	1.6	0.8
		1000	2.0	1.5	1.5	1.5	9.2	4.0	1.3	0.7
	0.1	200	1.9	1.8	1.1	0.9	17.5	6.2	1.9	0.9
		400	2.9	2.7	2.0	1.9	12.7	4.9	1.6	0.8
		1000	2.7	2.4	2.4	2.4	9.0	3.8	1.4	0.7
20	0.01	200	1.0	1.3	0.8	0.6	21.6	6.9	2.3	0.9
		400	0.8	0.7	0.8	1.0	15.8	7.3	1.8	0.8
		1000	1.8	1.7	1.1	0.7	15.2	4.0	1.7	0.7
	0.05	200	1.5	2.3	2.2	1.6	17.2	6.2	1.9	0.9
		400	2.0	2.9	2.2	1.8	12.7	4.9	1.6	0.8
		1000	2.3	3.3	2.5	2.1	9.0	3.8	1.4	0.7
	0.1	200	1.9	2.0	1.3	0.9	17.5	6.4	1.9	0.8
		400	4.1	2.5	2.6	1.4	12.6	5.0	1.7	0.7
		1000	4.6	4.9	3.3	3.1	9.0	3.9	1.4	0.7

Parameters: initial population size (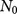); growth rate (*r*); carrying capacity (*K*); source population allele frequency (*Q*); minimal number of migrants from source population to founded population required to reach mean allele frequency *Q* at equilibrium (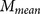); minimal number of migrants from source population to founded population required to reach 95% probability of presence of the allele at equilibrium (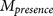).

While the simplified approximation summarized by equation 4 was far from an accurate evaluation of 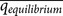 for migration rates below 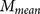, it did qualitatively describe the gradual increase in 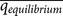 for migration rates below a threshold and stabilization of *Q* (S1–S9 Figs.). A better approximation was attained using a regression analysis with the model 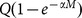
with unknown parameter *α* introduced to increase the goodness of fit of the model, shown in S1–S9 Figs., and with regression details presented in S3 Table. For the scenarios simulated, 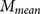 was estimated fairly well by equation 4 for rare alleles, but less so for more common alleles. The simplified approximation also showed migration thresholds for stabilization that were higher for common alleles, compared with rare alleles, a pattern similar to that shown by the 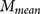 values from the simulations.

The migration thresholds when considering mean allele frequency and when considering the probability of allele presence were considerably different, as can be seen in Table 1. Although 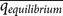 wa, s similar for migration rates above 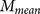, the actual probability distribution of the allele frequency revealed a different picture. For example, in Fig. 1, one can see very different distributions for the allele frequency at the equilibrium phase for two simulations with migration rates 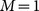 and 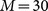 (other parameters: 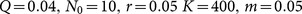). Specifically, the probability of allele presence with one migrant per generation was 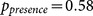, evident in the large discontinuous “jump” in the cumulative distribution function at allele frequency 0, while the probability for presence with 30 migrants per generation was much higher, with 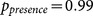. In contrast, the mean allele frequency values at equilibrium for these two migration rates were almost identical, with 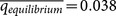 for 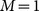 and 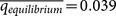 for 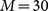.

**Figure 4:**
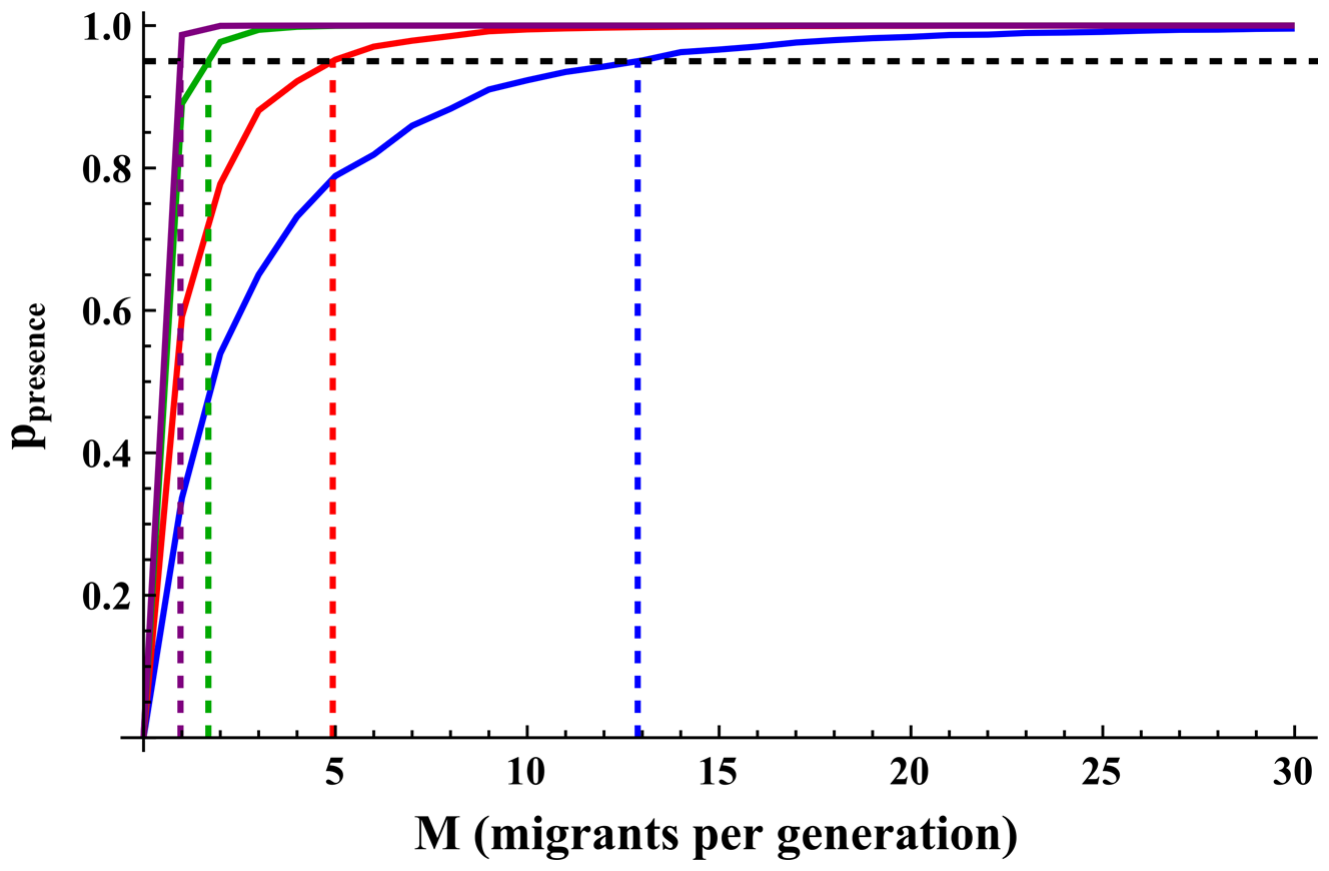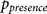 (probability of allele presence) for different migration rates. 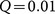
 in blue; 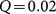 in red; 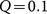 in green and 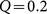 in purple. Horizontal dashed line show the 95% probability of allele presence threshold (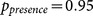) and vertical dashed lines show 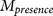 thresholds. Scenario parameters: 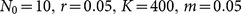. standard error of the mean was below 0.002 for all 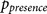 values and is therefore not indicated.

Fig. 4 shows the 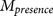 thresholds (number of migrants required to reach 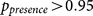) for four scenarios, and Table 1 summarizes the thresholds for all scenarios simulated. Evidently, 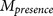 thresholds were very much affected by the source population allele frequency and were much higher for rare alleles than for more common alleles (an average of 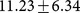 for 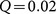 and 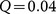 compared to an average of 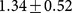 for 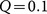 and 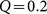). When comparing the 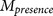 and 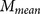 thresholds, for 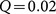, 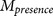 was much larger than 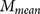 for all scenarios simulated (an average 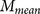 of 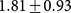 in contrast to an average 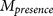 of 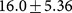), and for 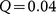 this was true for all scenarios but one (an average 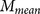 of 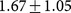 in contrast to an average 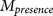 of 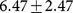). For the more common alleles, however, the two thresholds were generally similar (an average 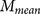 of 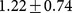 and an average 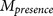 of 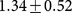 for 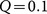 and 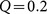 combined). In contrast to the 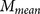 thresholds, the 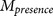 thresholds were generally lower for higher initial sizes, carrying capacities and growth rates; however, this was only evident for the rare alleles, while for the more common alleles, 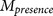 was not much affected by the change of scenario parameters.

### 3.1. Allele frequency spectrum

**Figure 5:**
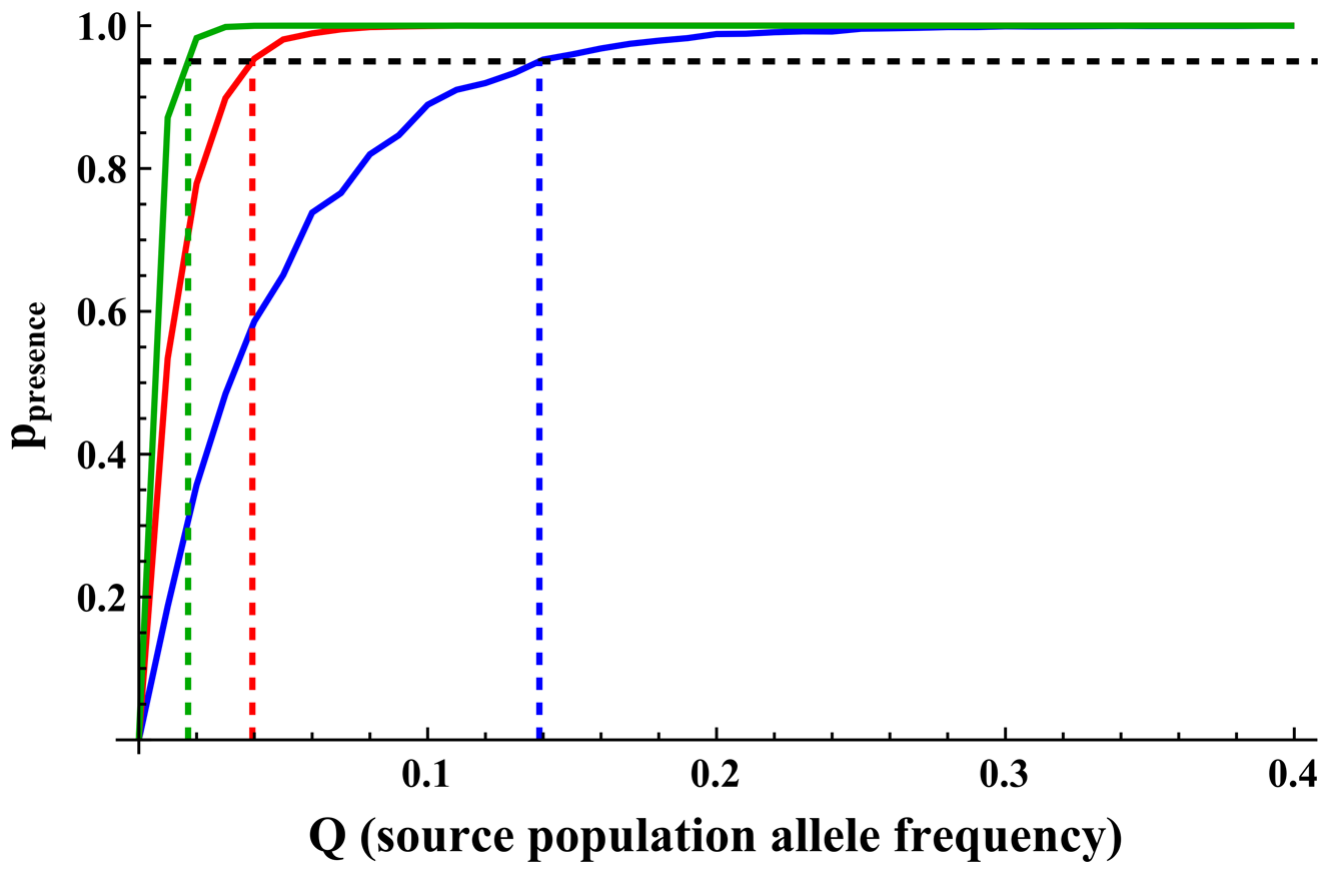
Probability of allele presence for different *Q* values (allele frequencies in source population). 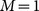
 in blue, 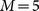 in red and 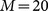 in green. Horizontal dashed line show the 95% probability of allele presence threshold (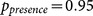) and vertical dashed lines show *Q_c_* thresholds (cut-off frequencies) for the different migration rates. Scenario parameters: 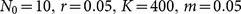.

Performing an analysis on polymorphic loci requires attention to different alleles with different frequencies, and not just a single allele. Fig. 5 shows how the cut-off frequency can be derived from the perspective of the allele frequency in the source population. For example, with 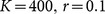 and 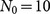, and one migrant per generation (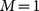), alleles of frequencies 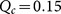 or higher are expected to be present in the founded population at equilibrium with a probability of 95% or higher, while alleles with lower frequencies are not. For the same demographic parameters, for 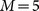 the cut-off frequency is 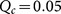, and for 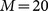 the cut-off frequency is 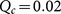.

Table 2 summarizes the cut-off frequencies and the proportion of allelic richness that is expected to be recovered, given the defined genetic goal of 95% probability of allele presence and the allele frequency spectra given in Fig. 2. Different migration rates result in different cut-off frequencies, and therefore have different impacts on the recovery potential of allelic richness. While the demographic parameters of the founded population seem to have little impact on the proportion of allelic richness expected to be recovered, the allele frequency spectrum of the source population has a major effect on the potential of recovery of alleles (*Q_c_* values in Table 2 do not vary much with different demographic parameters, but they do vary with different *θ* values). Allele frequency spectra that have few rare alleles (low *θ* values) have more alleles above the cut-off frequency and a higher potential for recovery of allelic richness. In contrast, allele frequencies with many rare alleles (high *θ* values) have many alleles below the cut-off frequency, alleles that are not expected to be recovered by gene flow and, as a result, show low potential for allelic richness recovery.

**Table 2:** Proportion of allelic richness recovered by gene flow and cut-off frequencies (*Q_c_*).

			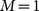				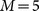				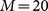			
				Proportion of allelic richness recovered				Proportion of allelic richness recovered				Proportion of allelic richness recovered		
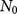	*r*	*K*	*Q_c_*	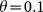	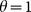	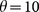	*Q_c_*	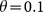	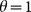	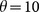	*Q_c_*	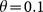	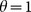	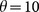
5	0.01	200	0.2	0.59	0.17	0.01	0.08	0.65	0.27	0.05	0.03	0.7	0.38	0.15
		400	0.16	0.6	0.2	0.01	0.05	0.67	0.33	0.09	0.03	0.7	0.38	0.15
		1000	0.18	0.6	0.19	0.01	0.04	0.68	0.35	0.11	0.04	0.68	0.35	0.11
	0.05	200	0.18	0.6	0.19	0.01	0.06	0.66	0.31	0.07	0.03	0.7	0.38	0.15
		400	0.2	0.59	0.17	0.01	0.06	0.66	0.31	0.07	0.03	0.7	0.38	0.15
		1000	0.17	0.6	0.19	0.01	0.06	0.66	0.31	0.07	0.03	0.7	0.38	0.15
	0.1	200	0.17	0.6	0.19	0.01	0.06	0.66	0.31	0.07	0.03	0.7	0.38	0.15
		400	0.16	0.6	0.2	0.01	0.06	0.66	0.31	0.07	0.03	0.7	0.38	0.15
		1000	0.13	0.62	0.22	0.02	0.05	0.67	0.33	0.09	0.02	0.72	0.42	0.2
10	0.01	200	0.16	0.6	0.2	0.01	0.05	0.67	0.33	0.09	0.02	0.72	0.42	0.2
		400	0.14	0.61	0.21	0.02	0.04	0.68	0.35	0.11	0.02	0.72	0.42	0.2
		1000	0.13	0.62	0.22	0.02	0.04	0.68	0.35	0.11	0.02	0.72	0.42	0.2
	0.05	200	0.17	0.6	0.19	0.01	0.05	0.67	0.33	0.09	0.02	0.72	0.42	0.2
		400	0.15	0.61	0.21	0.01	0.05	0.67	0.33	0.09	0.02	0.72	0.42	0.2
		1000	0.12	0.62	0.23	0.02	0.04	0.68	0.35	0.11	0.02	0.72	0.42	0.2
	0.1	200	0.16	0.6	0.2	0.01	0.05	0.67	0.33	0.09	0.02	0.72	0.42	0.2
		400	0.15	0.61	0.21	0.01	0.04	0.68	0.35	0.11	0.02	0.72	0.42	0.2
		1000	0.13	0.62	0.22	0.02	0.04	0.68	0.35	0.11	0.02	0.72	0.42	0.2
20	0.01	200	0.16	0.6	0.2	0.01	0.05	0.67	0.33	0.09	0.02	0.72	0.42	0.2
		400	0.14	0.61	0.21	0.02	0.04	0.68	0.35	0.11	0.02	0.72	0.42	0.2
		1000	0.12	0.62	0.23	0.02	0.04	0.68	0.35	0.11	0.02	0.72	0.42	0.2
	0.05	200	0.16	0.6	0.2	0.01	0.05	0.67	0.33	0.09	0.02	0.72	0.42	0.2
		400	0.14	0.61	0.21	0.02	0.04	0.68	0.35	0.11	0.02	0.72	0.42	0.2
		1000	0.13	0.62	0.22	0.02	0.04	0.68	0.35	0.11	0.02	0.72	0.42	0.2
	0.1	200	0.17	0.6	0.19	0.01	0.05	0.67	0.33	0.09	0.02	0.72	0.42	0.2
		400	0.14	0.61	0.21	0.02	0.04	0.68	0.35	0.11	0.02	0.72	0.42	0.2
		1000	0.12	0.62	0.23	0.02	0.04	0.68	0.35	0.11	0.02	0.72	0.42	0.2

Initial population size (*N_0_*); growth rate (*r*); carrying capacity (*K*); migrants per generation from source population to founded population (*M*); cut-off frequency (*Q_c_*); source population allele frequency spectrum (defined by equation 6) with given *θ* value.

## Discussion

The model follows a single allele that is subject to two evolutionary forces: gene flow and genetic drift. If the stochastic nature of genetic drift and migration pattern are ignored, equation 3 predicts that the mean allele frequency at equilibrium, 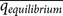, should converge to the allele frequency in the source population, *Q*. The observation that for the scenarios simulated, the mean allele frequency indeed converged to *Q* at equilibrium for high enough migration rates (Fig. 3 and S1–S9 Figs.) fits this deterministic prediction. For migration rates lower than the threshold (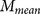), 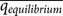 was lower and only qualitatively described by the simplified approximation given in equation 4. This lower 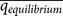 is due to the fact that the arrival times of the allele from the source population are long enough, compared to the time required for the allele to be lost, so that the allele is absent for significant periods from the population.

These last observations, and the simulation results of typical 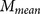 values of 0.5–2.5, might lead to the erroneous conclusion that in these scenarios, migration rates higher than one or two migrants per generation are enough to restore the loss of genetic diversity for most scenarios, as the mean allele frequency in the founded population is similar to that of the source population. This conclusion would be concordant with the analogous analysis of heterozygosity in a migration-drift island model that is exemplified in the OMPG rule (the One Migrant Per Generation rule states that one migrant per generation is an adequate gene flow rate to prevent loss of genetic diversity). The actual probability distributions (Fig. 1) and, specifically, the probability of allele presence (Fig. 4), reveal that this is not the case for allelic richness. In the scenarios simulated, the migration rates needed to reach the defined threshold of 95% probability of presence for relatively rare alleles are typically much higher than the migration rates needed for convergence of the mean frequency to *Q* (Table 1). This genetic goal (95% probability of allele presence [47]) directly addresses the question of the presence or the absence of the allele and is, therefore, a more appropriate statistical characteristic than the mean frequency of the allele for the analysis of allelic richness (since the expected value of allelic richness is, by definition, the probability of allele presence summed over all alleles and averaged over all loci).

In conservation, often there is concern to maintain as high as possible levels of genetic diversity, both of heterozygosity and allelic richness. Setting the genetic goal at 95% probability of presence, as defined above, is appropriate in conservation, since its interpretation, in terms of statistical confidence, is the “minimal allelic richness retained with a 95% confidence”, which could be applied in management programs or genetic assessments. The genetic goal affects the required rates of migration needed to maintain different levels of allelic richness, and lower genetic goals would show lower migration requirements (one could imagine, for example, a genetic goal line of 80% or 60% in Figs. 4 and 5). This can be used to give several thresholds with varying levels of confidence, which could be useful for decision making and management assessments.

### 4.1. Allele frequency spectrum analysis

Typically, it is not that the presence of a specific allele is of concern, but rather the presence of many alleles across many loci [51]. Therefore, a study of the allele frequency spectrum of the population is required (i.e., the number of alleles at different frequency intervals). From the perspective of the allele frequency spectrum, the questions shift to how many alleles will be recovered by gene flow following the founder effect, and what amount of allelic richness will be recovered from the source population and maintained in the founded population in a given scenario. We have used the same genetic goal of 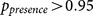 to obtain the ‘cut-off frequency’ *Q_c_* (the minimal allele frequency in the source population for which the allele is assumed to be present in the founded population at equilibrium) of a given scenario. *Q_c_* allows us to evaluate the effect gene flow has on the maintenance of allelic richness with regard to the entire frequency spectrum, and not just at the level of a single allele. For five migrants per generation, for example, *Q_c_* is lower than for one migrant per generation, meaning that rarer alleles are expected to be present in the population at equilibrium, and the population is expected to show higher allelic richness (Fig. 5 and Table 2).

In order to apply the framework presented here, an estimation of allele recovery that takes into account the allele frequency spectrum is needed. The cut-off thresholds can be used to calculate the portion of allelic richness that may potentially be recovered by gene flow in a given scenario by comparing them to an allele frequency spectrum, as shown in Fig. 5 (when comparing with an actual allele frequency rather than a theoretical one, the expected number and proportion of alleles above *Q_c_* should be derived directly from the allele frequency distribution of the population, and not from equation 5). This can give a quantitative estimate of the impact gene flow will have in a given scenario with a given allele frequency spectrum, as shown in Table 2. This may be particularly useful in conservation efforts aimed at restoring genetic diversity by active management (e.g., translocations), when genetic studies of source populations can provide an allele frequency spectrum (e.g., by averaging allele spectra from several loci), and management strategies can be evaluated by conducting simulations, in the presented framework, for appropriate scenarios.

The results emphasize the importance of taking into account the source population's allele frequency spectrum when evaluating allelic richness recovery by gene flow, as this parameter had the greatest impact on allelic richness recovery. Most allele frequency spectrums are “L-shaped spectrums” - they consist of many rare alleles and few common alleles [48]. Since rare alleles are the ones most susceptible to loss through founder effect, they are the ones that mainly need to be considered when estimating genetic diversity. This abundance of rare alleles in many populations is not represented well by heterozygosity measures and is better addressed by allelic richness [2].

The analysis presented here estimates the potential of allele recovery following a founder effect, but does not directly consider the founder event itself. The framework has been generalized to include a simple founder event (S3 Appendix); however, this entails additional simulations to account for initial conditions different than 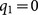. Nevertheless, for scenarios with low enough *Q_c_* values, 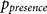 is a good approximation for the probability of presence including the founder event (since for low *Q* values the allele is likely to be lost in the founder event or soon after, see details in S3 Appendix). The results obtained from scenarios with low *Q_c_* are thus an approximation of the proportion of allelic richness expected to be retained from founder event to migration-drift balance.

Although analyzing the results from simulations that track single alleles at different allele frequencies in the source population in light of an allele frequency spectrum, as presented here, provides a reasonably simple approach for the evaluation of allelic richness recovery, it does have its limitations. One important limitation is the fact that the probabilities of allele presence of different alleles are not independent, while the cut-off frequency, used to calculate the number and proportion of allele recovery, assumes independence. Thus, results should be used as an approximation of the expected allele recovery, and more computationally complex simulations of multiple alleles with frequencies taken from an appropriate allele frequency spectrum are needed to obtain more accurate results for specific scenarios.

### 4.2. The OMPG rule and allelic richness

Conservation and management programs increasingly address genetic issues, as small, vulnerable populations are susceptible to genetic diversity loss, which might negatively impact their status [1]. Management programs usually include some reference to general rules and try to put them in the context of the scenario in question. Since F-statistics are amongst the most widely used frameworks for genetic differentiation [52], they are the source of many conservation-genetic rules and guidelines [53].

One such conservation rule is the OMPG rule [54]. This regularly used rule [21] is based on the results of an F-statistics derivation of the infinite islands model that implies that only the product of genetic drift and migration rate (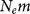) need be taken into account, where *N_e_* is the local variance effective population size. The OMPG rule states that one effective migrant per generation (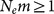) is enough for conservation purposes. While the original papers concerning the OMPG rule emphasize the limitation of the rule, and that its interpretation is limited to the equilibrium state of the mean allele frequency (and not the presence\absence of alleles) in an island model of migration, the OMPG rule is extensively used in the conservation and management of captive populations. The model and framework presented in this paper show that the ‘probability of allele presence’ (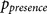), which is the relevant statistic for assessing allelic richness, is not adequately addressed by the OMPG rule. The number of migrants required for allelic richness maintenance, at least when founding events are considered, depends on the specific parameters of the scenario (e.g., allele frequency spectrum, demographic parameters), and in some cases, for instance when source population allele frequency spectra with many rare alleles are concerned, the migration rate required for the presence of the allele at equilibrium could be much higher than just one migrant per generation.

This analysis points out, once again, that the OMPG rule has limitations (for limitations from other perspectives, see [21], [55]). More specifically, its application should be reserved for cases in which low heterozygosity is a concern. Maintenance of heterozygosity might be achieved with one migrant per generation, but this can be too low a migration rate for allelic richness maintenance. Following a founder effect, heterozygosity should be a concern for the period immediately following the event, but allelic richness should be more important for the long-term evolutionary potential of the population [2], [12], and the OMPG rule should be used with this distinction in mind.

### 4.3. Allelic richness vs. heterozygosity

Allelic richness and heterozygosity form the basis of the two most commonly used measures of genetic diversity, but heterozygosity is applied much more regularly. While a third group of locus-level diversity measures, based on Shannon's index, has been suggested and has recently seen some development [56], the evolutionary interpretation of these measures is still unclear [57], and they are not yet in common use. Allelic richness measures are essential to our understanding of the aspect of a population's genetic diversity pertaining to the long term evolutionary potential of the population [2], [13]–[15], [17]. The differences between the measures, in regards to treatment of rare and common alleles, are perhaps more apparent when considering them as two diversity indices. Allelic richness is a 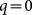 diversity measure (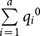 where *a* is the number of alleles and 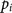 are the allele frequencies), and heterozygosity is the Geni-Simpson index, which has a 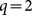 diversity (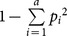)[58], [59]. The order of the diversity index, *q*, indicates the sensitivity of the measure to common and rare alleles, where a 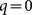 diversity is completely insensitive to allele frequencies and, therefore, favors rare alleles, and 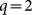 diversity favors common alleles (with a 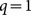 diversity, Shannon's index, rare and common alleles are proportionally weighted)[56], [58]. Allelic richness, therefore, quantifies the actual number of alleles, while heterozygosity can be seen to quantify 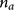, the “effective number of alleles” (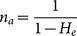) – the number of alleles expected in a population with the same heterozygosity but with allele frequencies distributed equally[12], [13], [60], [61].

In this paper, we suggest a genetic goal of ‘95% probability of allele presence’ that emphasizes the importance of the presence of alleles over their frequency, as is appropriate for a conservative allelic richness evaluation (lower genetic goals may be used to give evaluations with lower confidence of allele presence). The presence of alleles is indicative of the potential of selection to act upon an allele and, thus, relates directly to the evolutionary potential of the population[13]. This genetic goal can be used in analysis of stochastic models and in combination with an allele frequency spectrum to provide predictions for allelic richness under different ecological scenarios, as shown in the model presented here.

While F-statistics provide a framework for analyzing heterozygosity dynamics, our understanding of allelic richness dynamics is limited. Allelic richness, which emphasizes the number of alleles over their frequencies, is affected by various parameters, such as migration rates, allele frequency and demographic parameters, as demonstrated by the model results. However, heterozygosity, which focuses on allele frequencies and thus is insensitive to rare alleles, is affected differently by different parameters in similar systems [2], [16], [18], [41], [62]. Thus, an allelic richness evaluation requires different considerations than that of an F-statistics framework, as demonstrated by the comparison of the OMPG rule with the simulation results. With the ecological and evolutionary consequences of heterozygosity versus allelic richness in mind, both measures should be considered in conservation and management efforts aiming at maintaining genetic diversity.

## Supporting Information

S1 Appendix Migration patterns and allelic diversity.
(DOCX)

S2 Appendix Code for the simulations.
(NB) Click here for additional data file.

S3 Appendix Generalization of the framework to include a simple one-generation founder event.
(DOCX)

S1 FigMean allele frequencies at equilibrium (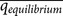) as a function of the number of migrants per generation (*M*). Solid lines indicate the estimation of the mean allele frequency (equation 4). Dashed lines indicate 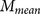 thresholds. Dashed-dotted lines indicate regression analysis results for the model 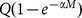; details in S3 Table. 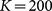 in blue; 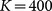 in red; 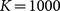 in blue. A) 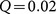; B) 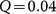; C) 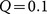; D) 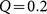. Scenario parameters: 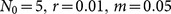. Error bars indicate the standard error of the mean.(TIFF)

S2 FigMean allele frequencies at equilibrium (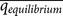) as a function of the number of migrants per generation (*M*). Solid lines indicate the estimation of the mean allele frequency (equation 4). Dashed lines indicate 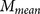 thresholds. Dashed-dotted lines indicate regression analysis results for the model 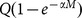; details in S3 Table. 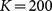 in blue; 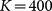 in red; 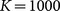 in blue. A) 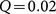; B) 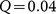; C) 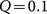; D) 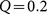. Scenario parameters: 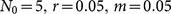. Error bars indicate the standard error of the mean.(TIFF)

S3 FigMean allele frequencies at equilibrium (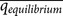) as a function of the number of migrants per generation (*M*). Solid lines indicate the estimation of the mean allele frequency (equation 4). Dashed lines indicate 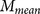 thresholds. Dashed-dotted lines indicate regression analysis results for the model 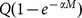; details in S3 Table. 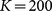 in blue; 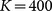 in red; 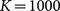 in blue. A) 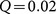; B) 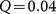; C) 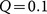; D) 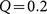. Scenario parameters: 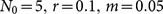. Error bars indicate the standard error of the mean.(TIFF)

S4 FigMean allele frequencies at equilibrium (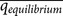) as a function of the number of migrants per generation (*M*). Solid lines indicate the estimation of the mean allele frequency (equation 4). Dashed lines indicate 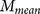 thresholds. Dashed-dotted lines indicate regression analysis results for the model 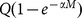; details in S3 Table. 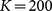 in blue; 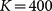 in red; 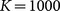 in blue. A) 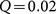; B) 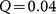; C) 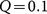; D) 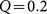. Scenario parameters: 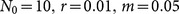. Error bars indicate the standard error of the mean.(TIFF)

S5 FigMean allele frequencies at equilibrium (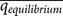) as a function of the number of migrants per generation (*M*). Solid lines indicate the estimation of the mean allele frequency (equation 4). Dashed lines indicate 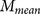 thresholds. Dashed-dotted lines indicate regression analysis results for the model 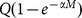; details in S3 Table. 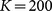 in blue; 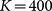 in red; 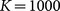 in blue. A) 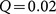; B) 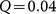; C) 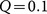; D) 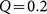. Scenario parameters: 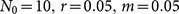. Error bars indicate the standard error of the mean.(TIFF)

S6 FigMean allele frequencies at equilibrium (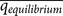) as a function of the number of migrants per generation (*M*). Solid lines indicate the estimation of the mean allele frequency (equation 4). Dashed lines indicate 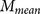 thresholds. Dashed-dotted lines indicate regression analysis results for the model 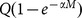; details in S3 Table. 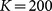 in blue; 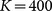 in red; 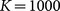 in blue. A) 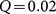; B) 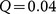; C) 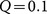; D) 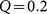. Scenario parameters: 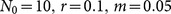. Error bars indicate the standard error of the mean.(TIFF)

S7 FigMean allele frequencies at equilibrium (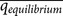) as a function of the number of migrants per generation (*M*). Solid lines indicate the estimation of the mean allele frequency (equation 4). Dashed lines indicate 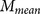 thresholds. Dashed-dotted lines indicate regression analysis results for the model 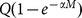; details in S3 Table. 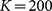 in blue; 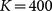 in red; 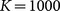 in blue. A) 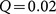; B) 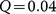; C) 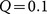; D) 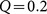. Scenario parameters: 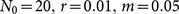. Error bars indicate the standard error of the mean.(TIFF)

S8 FigMean allele frequencies at equilibrium (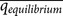) as a function of the number of migrants per generation (*M*). Solid lines indicate the estimation of the mean allele frequency (equation 4). Dashed lines indicate 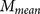 thresholds. Dashed-dotted lines indicate regression analysis results for the model 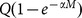; details in S3 Table. 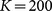 in blue; 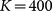 in red; 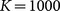 in blue. A) 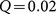; B) 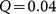; C) 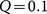; D) 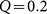. Scenario parameters: 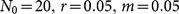. Error bars indicate the standard error of the mean.(TIFF)

S9 FigMean allele frequencies at equilibrium (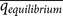) as a function of the number of migrants per generation (*M*). Solid lines indicate the estimation of the mean allele frequency (equation 4). Dashed lines indicate 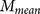 thresholds. Dashed-dotted lines indicate regression analysis results for the model 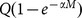; details in S3 Table. 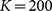 in blue; 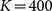 in red; 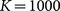 in blue. A) 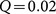; B) 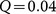; C) 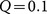; D) 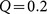. Scenario parameters: 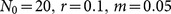. Error bars indicate the standard error of the mean.(TIFF)

S1 Table

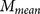
 and 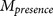 thresholds for different scenarios with deterministic migration pattern. Parameters: initial population size (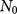); growth rate (*r*); carrying capacity (*K*); source population allele frequency (*Q*); minimal number of migrants from source population to founded population required to reach mean allele frequency *Q* at equilibrium (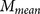); minimal number of migrants from source population to founded population required to reach 95% probability of presence of the allele at equilibrium (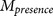).(DOCX)

S2 TableProportion of allelic richness recovered by gene flow and cut-off frequencies (*Q_c_*) for deterministic migration pattern. Initial population size (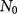); growth rate (*r*); carrying capacity (*K*); migrants per generation from source population to founded population (*M*); cut-off frequency (*Q_c_*); source population allele frequency spectrum (defined by equation 6) with given θ value.(DOCX)

S3 TableFitted regression models for 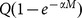 for mean allele frequency at equilibrium. Curves shown in S1-S9 Figs.(DOCX)
