# Correction: Allelic Richness following Population Founding Events—A Stochastic Modeling Framework Incorporating Gene Flow and Genetic Drift

**DOI:** 10.1371/journal.pone.0119663

**Published:** 2015-03-20

**Authors:** The PLOS ONE Staff

There is an error in the caption of Fig. 1; the words “red” and “blue” should be switched. Please see the correct caption here.

**Fig 1:**
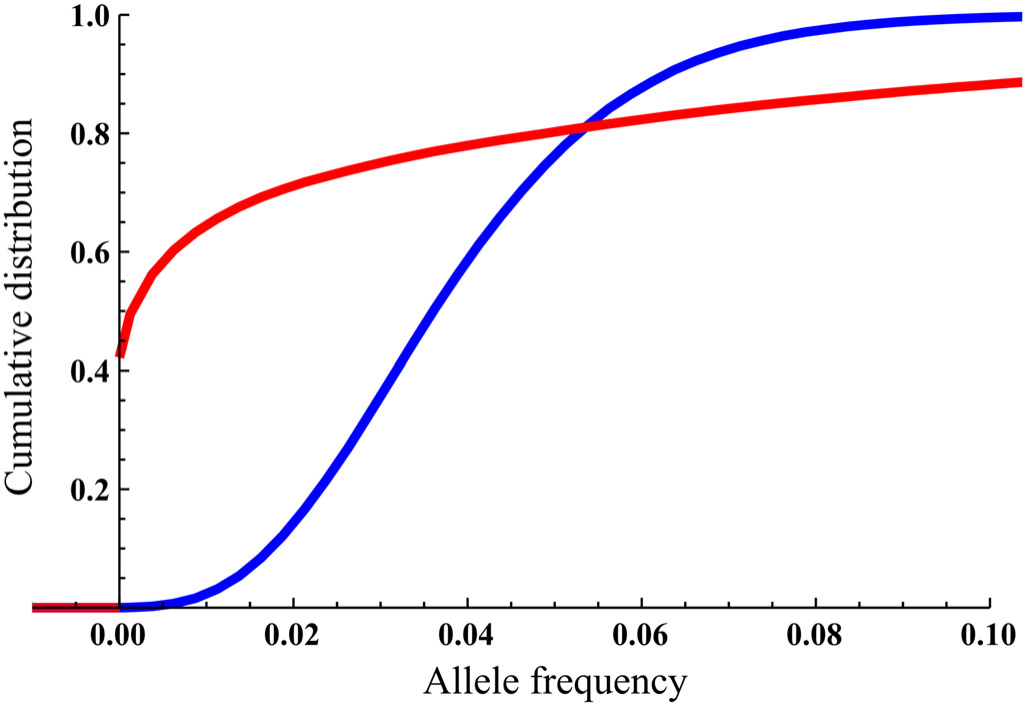
Cumulative density functions for the allele frequency at the equilibrium phase. *M* = 1 migrants in red, *M* = 30 migrants in blue; scenario parameters *Q* = 0.04, *N*_0_ = 10, *K* = 400, *r* = 0.05, *m* = 0.05. The jump at allele frequency 0 for *M* = 1 is due to the low *p_presence_* for this scenario.
