# Supplementary material for: Allelic Richness following Population Founding Events – A Stochastic Modeling Framework Incorporating Gene Flow and Genetic Drift
Source: PLoS One. 2014 Dec 19;9(12):e115203. doi: 10.1371/journal.pone.0115203 (PMC4272294; doi:10.1371/journal.pone.0115203)
Supplement: S1 Appendix — Migration patterns and allelic diversity. (DOCX) [file pone.0115203.s001.docx]

**Appendix S1**

*Migration patterns and allelic diversity*

Different migration patterns might affect the genetic diversity of the populations in different ways, even when the mean number of migrants is the same (Gaggiotti & Smouse 1996). Such migration patterns could be deterministic (i.e., a constant number of migrants per generation), or they could be characterized by some other distribution. A migration pattern that is appropriate for modeling many natural phenomena, such as when it could be assumed that there is a constant probability for each individual to migrate and the population is large enough, is represented by a Poisson distribution. A deterministic migration could be appropriate, for example, when migration is artificially created by a translocation program, in which migration rates can be controlled and monitored. Various other patterns could be appropriate for other scenarios.

While it has been shown that migration patterns have an effect on the heterozygosity of populations (Gaggiotti & Smouse 1996), it has not been demonstrated for allelic richness. In order to address this issue, simulations have been performed to understand whether deterministic migration has a different effect on the recovery of alleles in a founder scenario than do migration patterns with a Poisson distribution (as used in the simulation model in the main text), when both have the same mean. This has been done using the same methods as described in the Methods Section, but with the number of migrants taken to be constant. The results are presented in Tables S1 and S2.

The probabilities of allele presence ($p_{presence}$) for different scenarios were almost identical in both migration patterns, and this condition is also evident in the $M_{presence}$ and *Q_c_* thresholds (Tables S1 and S2). As a result, the proportion of allelic richness expected to be recovered by gene flow is almost identical for equivalent scenarios. These results indicate that the migration pattern has little effect on allelic richness in founder scenarios. However, since this analysis covers only a small number of scenarios, further study of the effect of migration patterns on allelic richness vs. heterozygosity is needed in order to understand what genetic effects are induced by migration patterns.

Literature Cited

Gaggiotti, O. E., and P. E. Smouse. 1996. Stochastic migration and maintenance of genetic variation in sink populations. American Naturalist **:**919-945.
