# Supplementary material for: Allelic Richness following Population Founding Events – A Stochastic Modeling Framework Incorporating Gene Flow and Genetic Drift
Source: PLoS One. 2014 Dec 19;9(12):e115203. doi: 10.1371/journal.pone.0115203 (PMC4272294; doi:10.1371/journal.pone.0115203)
Supplement: S3 Appendix — Generalization of the framework to include a simple one-generation founder event. (DOCX) [file pone.0115203.s003.docx]

**Appendix S3 – Generalization of the framework to include a simple one-generation founder event**

The framework can be generalized to include a simple one-generation founder event, and thus results in the form of Tables 1-3 would show the proportion of allelic diversity expected to be retained following the founder event and population expansion until migration-drift balance is reached. The generalization is based on similar simulations as described in the main text, only including different initial conditions for the model. This can account for instances in which the allele was not lost during the founding event, but rather was present in the newly founded population at a certain frequency.

When using the model formulated in equation 2 and an initial condition $q_{1}=x$, the simulation can be performed in the same manner as presented in the main text. The analysis can be repeated, and a new parameter $p_{presence}$ can be computed. For initial condition $q_{1}=x$, we will denote the $p_{presence}$ value obtained using the simulation framework as $p_{presence}(x)$(the $p_{presence}$ value described in the main text is $p_{presence}(0)$ under this notation). It is possible to calculate the probability that, given a simple one-generation founder event with initial population size $N_{0}$, the initial frequency of the allele is $q_{1}=\frac{i}{2N_{0}}$ using a binomial distribution:

$Prob(q_{1}=\frac{i}{2N_{0}})=\left(\begin{matrix} 2N_{0} \\ i \end{matrix}\right)Q^{i}(1-Q)^{2N_{0}-1}$ .

Thus, following Denniston [1], the probability of the allele’s presence at equilibrium, taking into consideration the founder event, denoted as $\overline{p_{presence}}$, can be calculated under the presented framework as:

$\overline{p_{presence}}=\sum_{i=0}^{2N_{0}} Prob(q_{1}=\frac{i}{2N_{0}})p_{presence}(\frac{i}{2N_{0}})$ .

$p_{presence}(\frac{i}{2N_{0}})$ values are attained through $2N_{0}+1$simulations, such as the ones described in the main text, with different initial conditions.

Note that for $Q\ll\frac{1}{2N_{0}}$, the allele is most likely lost during the founder event (i.e., $Prob(q_{1}=0)\approx1$), and therefore

$\overline{p_{presence}}\approx p_{presence}(0)$ .

For higher *Q* values, but still low values not much larger than $\frac{1}{2N_{0}}$, $\overline{p_{presence}}$ is dominated by the first few terms of equation 8. Thus, only simulations with initial conditions $q_{1}=\frac{1}{2N_{0}}$ for small *i* values need to be considered. For these values, it is reasonable to assume that in most individual simulation runs, the allele will be lost due to drift in the first few generations of the simulation, before new alleles arrive from the source population (the expected time to reappearance of the allele is $\frac{1}{2MQ}$, which is long for low *Q* values). Thus, most of these runs will converge with runs that effectively begin with $q_{1}=0$, although for different demographic functions (same growth rate *r* and carrying capacity *K* values, only different initial population size $N_{0}$values). Therefore, for the first few relevant terms in equation 8, $p_{presence}\left(\frac{1}{2N_{0}}\right)\approx p_{presence}(0)$, and again equation 9 holds.

The analysis presented in the main text (such as presented in Table 1 and Table 2) can consequently be used as an approximation for the actual proportion of alleles retained, taking into consideration the founder effect, only given that *Q_c_* (the cut-off frequency) is $Q_{c}<\frac{1}{2N_{0}}$ or at least is not much larger than $\frac{1}{2N_{0}}$. Otherwise, further simulations are needed to attain more $p_{presence}(x)$ for as many terms needed in equation 8. For *Q* values close to 0.5, probably all terms are needed for a good approximation. In any case, in order to increase the accuracy of the estimation, calculating all $p_{presence}(x)$ is recommended.

The generalization of the framework described above considers only a simple one-generation founder event, but there are more complicated descriptions of population founder events. While equation 7 does not hold for founder events other than a simple one-generation founder event, if $Prob(q_{1}=\frac{i}{2N_{0}})$ for all $0\leq i\leq2N_{0}$ can be attained using other means (e.g., mathematical formulations or simulations), then equation 8 will still hold, and the framework can accommodate these generalizations as well. Such probabilities have been described for more general types of founder events by other authors (e.g. [2–10]).

**References**

1. Denniston C (1978) Small population size and genetic diversity: implications for endangered species. In: Temple SA, editor. Endangered birds: management techniques for preserving threatened species. Madison, WI: University of Wisconsin Press. pp. 281–289.

2. Watterson GA (1984) Allele frequencies after a bottleneck. Theor Popul Biol 26: 387–407.

3. Griffiths RC, Tavare S (1994) Sampling theory for neutral alleles in a varying environment. Philos Trans R Soc LondonSeries B Biol Sci 344: 403–410.

4. England PR, Osler GHR, Woodworth LM, Montgomery ME, Briscoe DA, et al. (2003) Effects of intense versus diffuse population bottlenecks on microsatellite genetic diversity and evolutionary potential. Conserv Genet 4: 595–604.

5. Kuo C, Janzen FJ (2003) bottlesim: a bottleneck simulation program for long lived species with overlapping generations. Mol Ecol Notes 3: 669–673.

6. Cornuet JM, Luikart G (1996) Description and power analysis of two tests for detecting recent population bottlenecks from allele frequency data. Genetics 144: 2001–2014.

7. Maruyama T, Fuerst P (1984) Population bottlenecks and nonequilibrium models in population genetics. I. Allele numbers when populations evolve from zero variability. Genetics 108: 745–763.

8. Maruyama T, Fuerst P (1985) Population bottlenecks and nonequilibrium models in population genetics. II. Number of alleles in a small population that was formed by a recent bottleneck. Genetics 111: 675–689.

9. Watterson G (1989) The neutral alleles model with bottlenecks‏. In: Feldman MW, editor. Mathematical Evolutionary Theory. Princeton, New Jersey: Princeton University Press. pp. 26–40.

10. Ewens W (1972) The sampling theory of selectively neutral alleles. Theor Popul Biol 112: 87–112.
