# Supplementary figures and images for: Allelic Richness following Population Founding Events – A Stochastic Modeling Framework Incorporating Gene Flow and Genetic Drift
Source: PLoS One. 2014 Dec 19;9(12):e115203. doi: 10.1371/journal.pone.0115203 (PMC4272294; doi:10.1371/journal.pone.0115203)

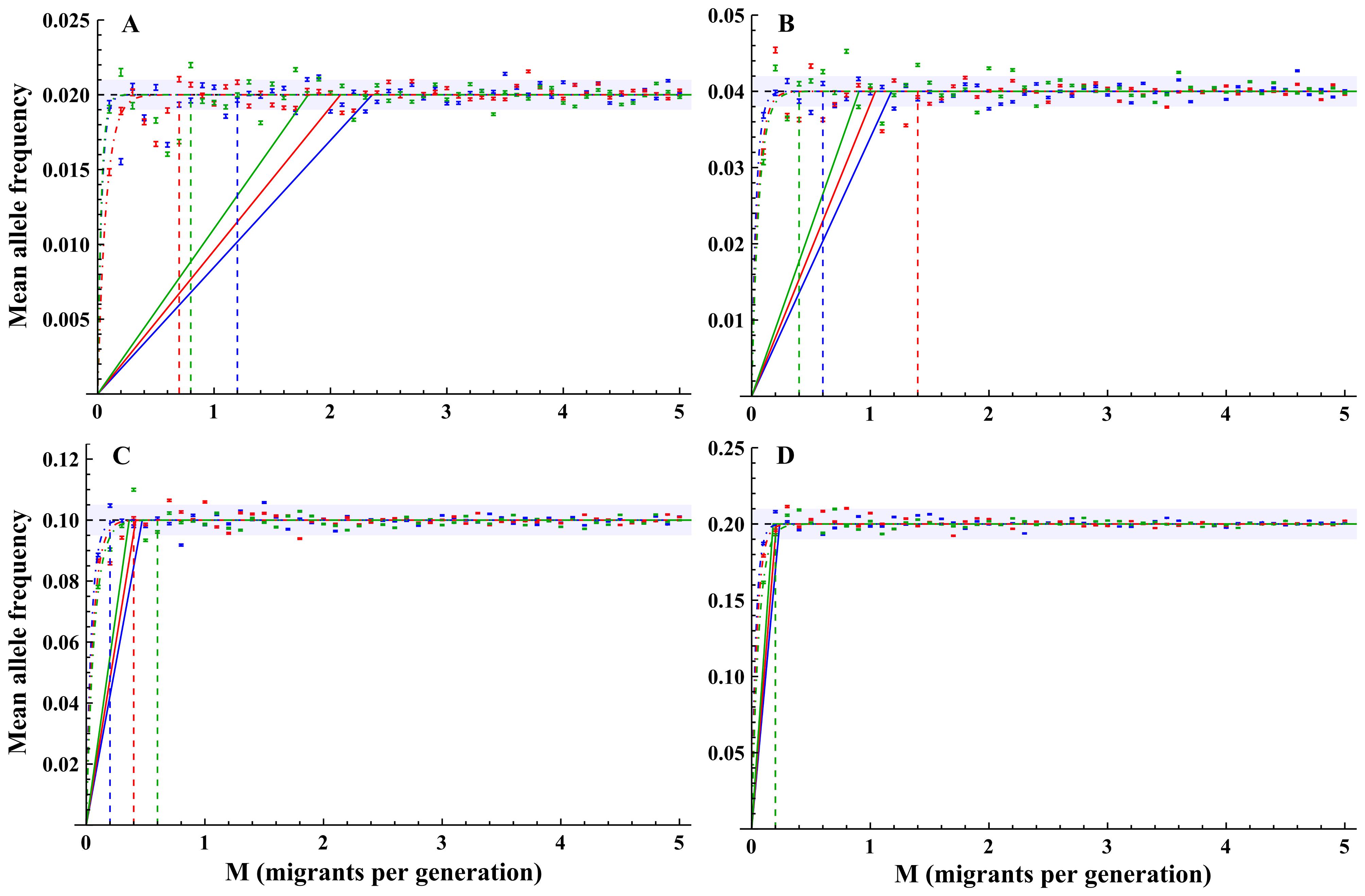

Supplement: S1 Fig — Mean allele frequencies at equilibrium () as a function of the number of migrants per generation (M). Solid lines indicate the estimation of the mean allele frequency (equation 4). Dashed lines indicate thresholds. Dashed-dotted lines indicate regression analysis results for the model ; details in S3 Table. in blue; in red; in blue. A) ; B) ; C) ; D) . Scenario parameters: . Error bars indicate the standard error of the mean. (TIFF) [file pone.0115203.s004.tiff]

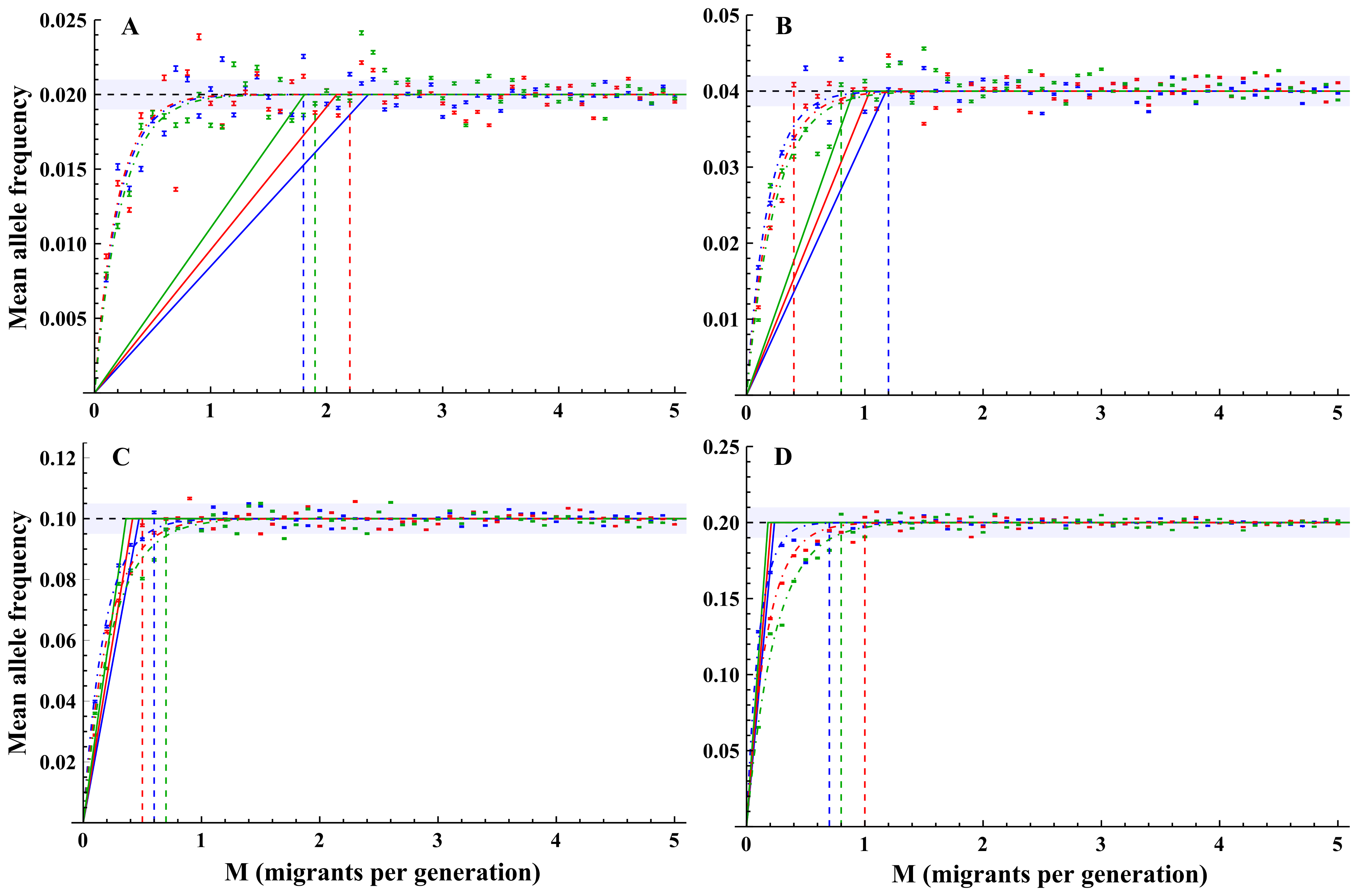

Supplement: S2 Fig — Mean allele frequencies at equilibrium () as a function of the number of migrants per generation (M). Solid lines indicate the estimation of the mean allele frequency (equation 4). Dashed lines indicate thresholds. Dashed-dotted lines indicate regression analysis results for the model ; details in S3 Table. in blue; in red; in blue. A) ; B) ; C) ; D) . Scenario parameters: . Error bars indicate the standard error of the mean. (TIFF) [file pone.0115203.s005.tiff]

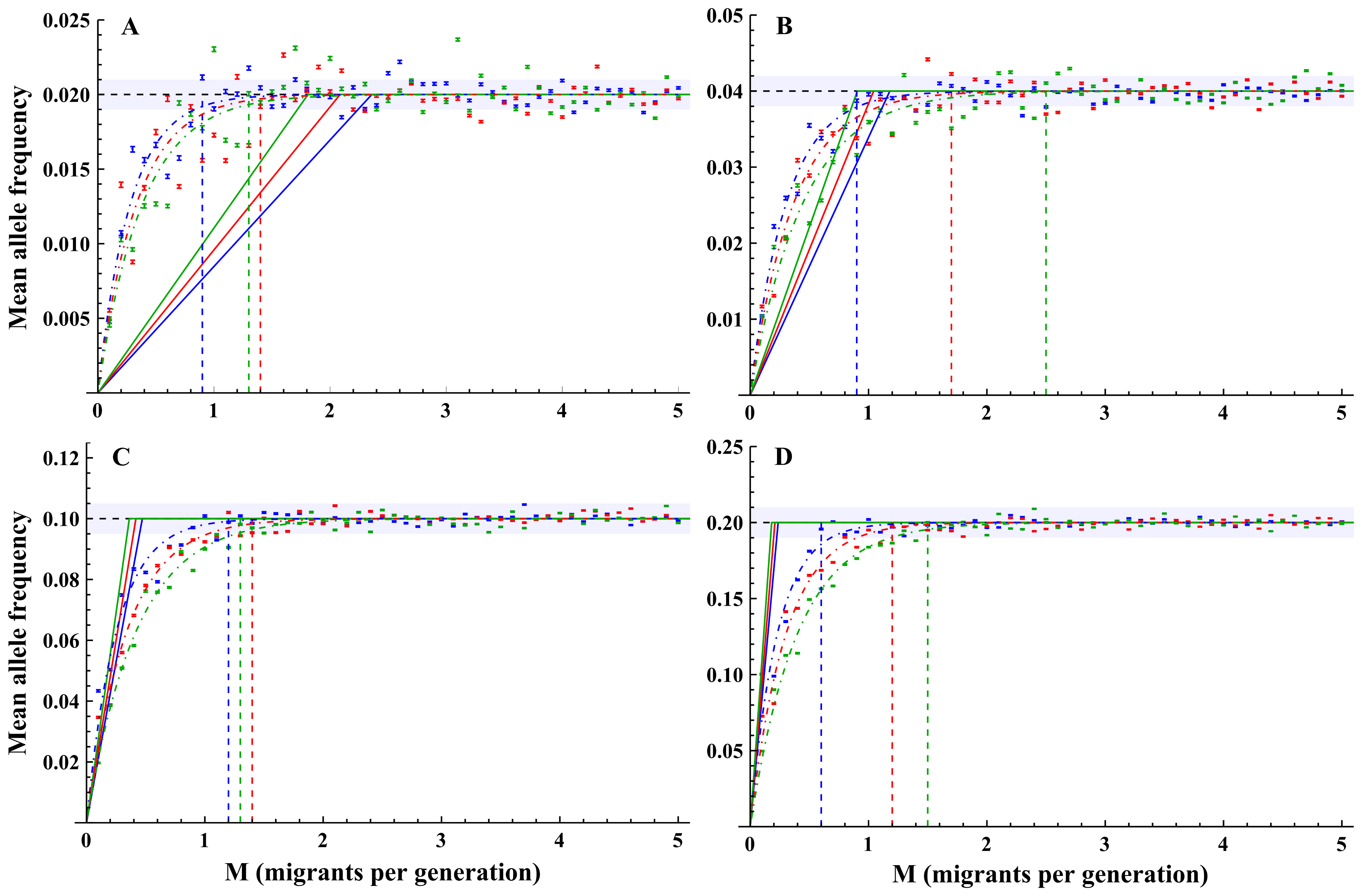

Supplement: S3 Fig — Mean allele frequencies at equilibrium () as a function of the number of migrants per generation (M). Solid lines indicate the estimation of the mean allele frequency (equation 4). Dashed lines indicate thresholds. Dashed-dotted lines indicate regression analysis results for the model ; details in S3 Table. in blue; in red; in blue. A) ; B) ; C) ; D) . Scenario parameters: . Error bars indicate the standard error of the mean. (TIFF) [file pone.0115203.s006.tiff]

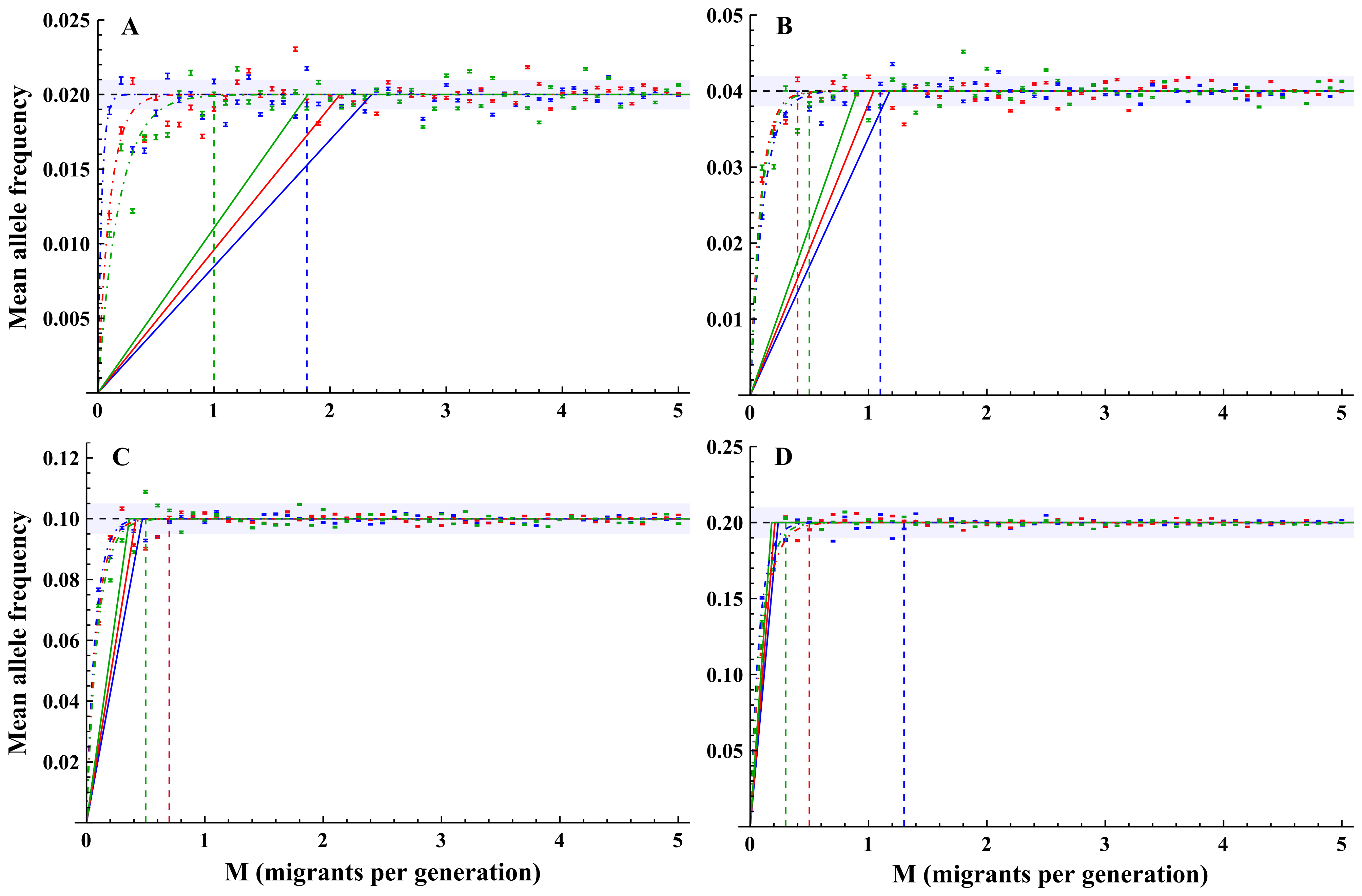

Supplement: S4 Fig — Mean allele frequencies at equilibrium () as a function of the number of migrants per generation (M). Solid lines indicate the estimation of the mean allele frequency (equation 4). Dashed lines indicate thresholds. Dashed-dotted lines indicate regression analysis results for the model ; details in S3 Table. in blue; in red; in blue. A) ; B) ; C) ; D) . Scenario parameters: . Error bars indicate the standard error of the mean. (TIFF) [file pone.0115203.s007.tiff]

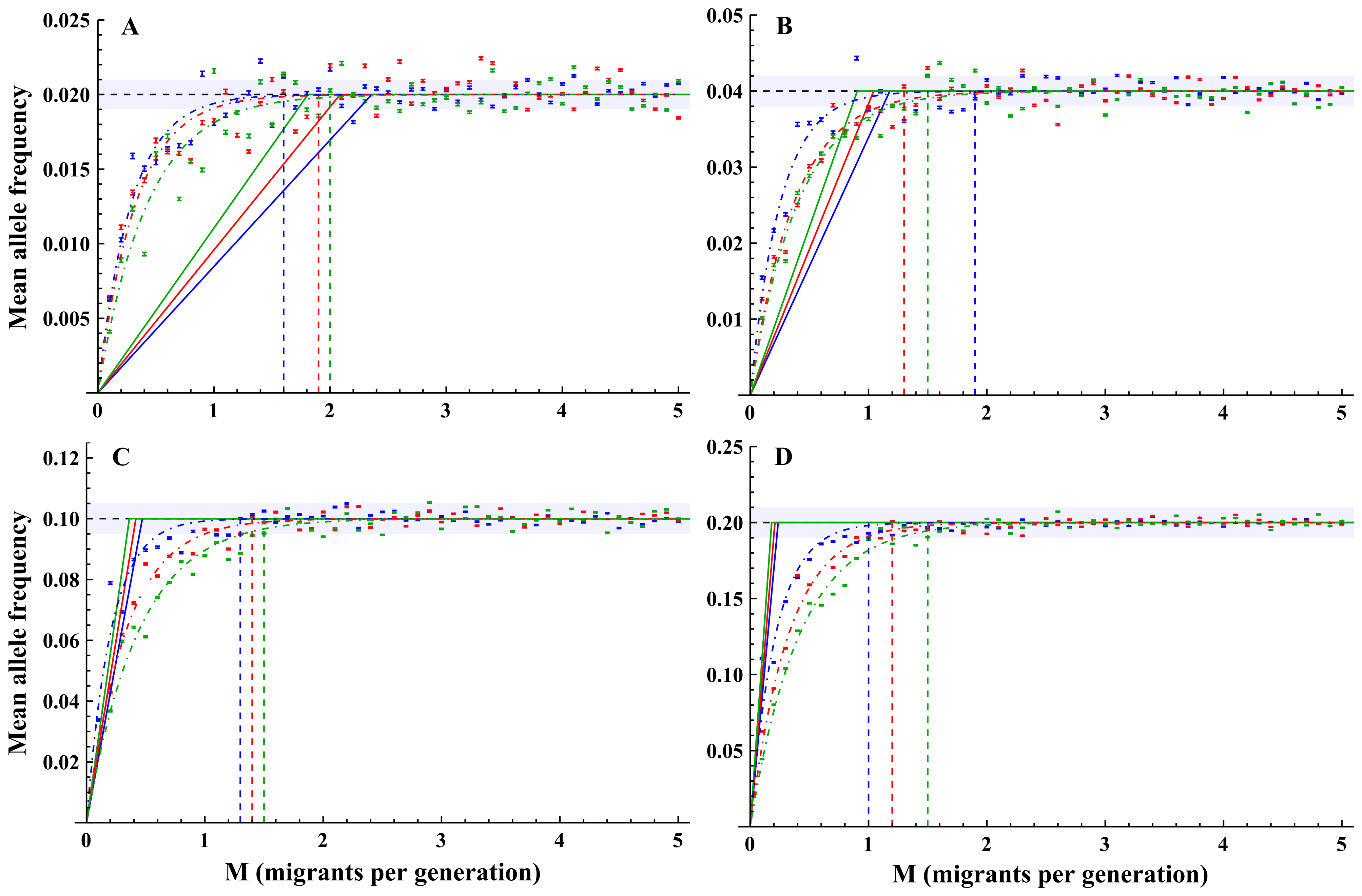

Supplement: S5 Fig — Mean allele frequencies at equilibrium () as a function of the number of migrants per generation (M). Solid lines indicate the estimation of the mean allele frequency (equation 4). Dashed lines indicate thresholds. Dashed-dotted lines indicate regression analysis results for the model ; details in S3 Table. in blue; in red; in blue. A) ; B) ; C) ; D) . Scenario parameters: . Error bars indicate the standard error of the mean. (TIFF) [file pone.0115203.s008.tiff]

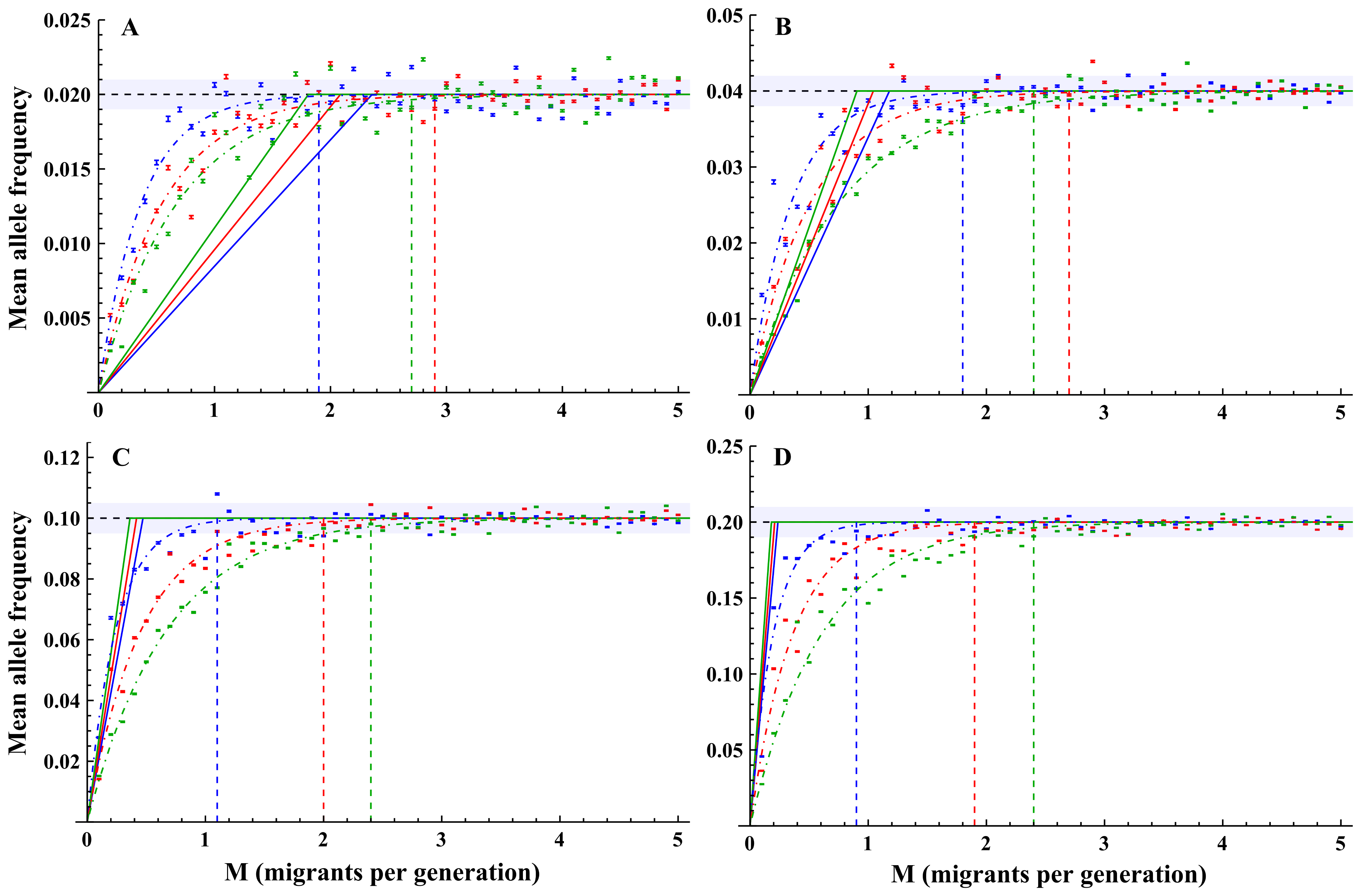

Supplement: S6 Fig — Mean allele frequencies at equilibrium () as a function of the number of migrants per generation (M). Solid lines indicate the estimation of the mean allele frequency (equation 4). Dashed lines indicate thresholds. Dashed-dotted lines indicate regression analysis results for the model ; details in S3 Table. in blue; in red; in blue. A) ; B) ; C) ; D) . Scenario parameters: . Error bars indicate the standard error of the mean. (TIFF) [file pone.0115203.s009.tiff]

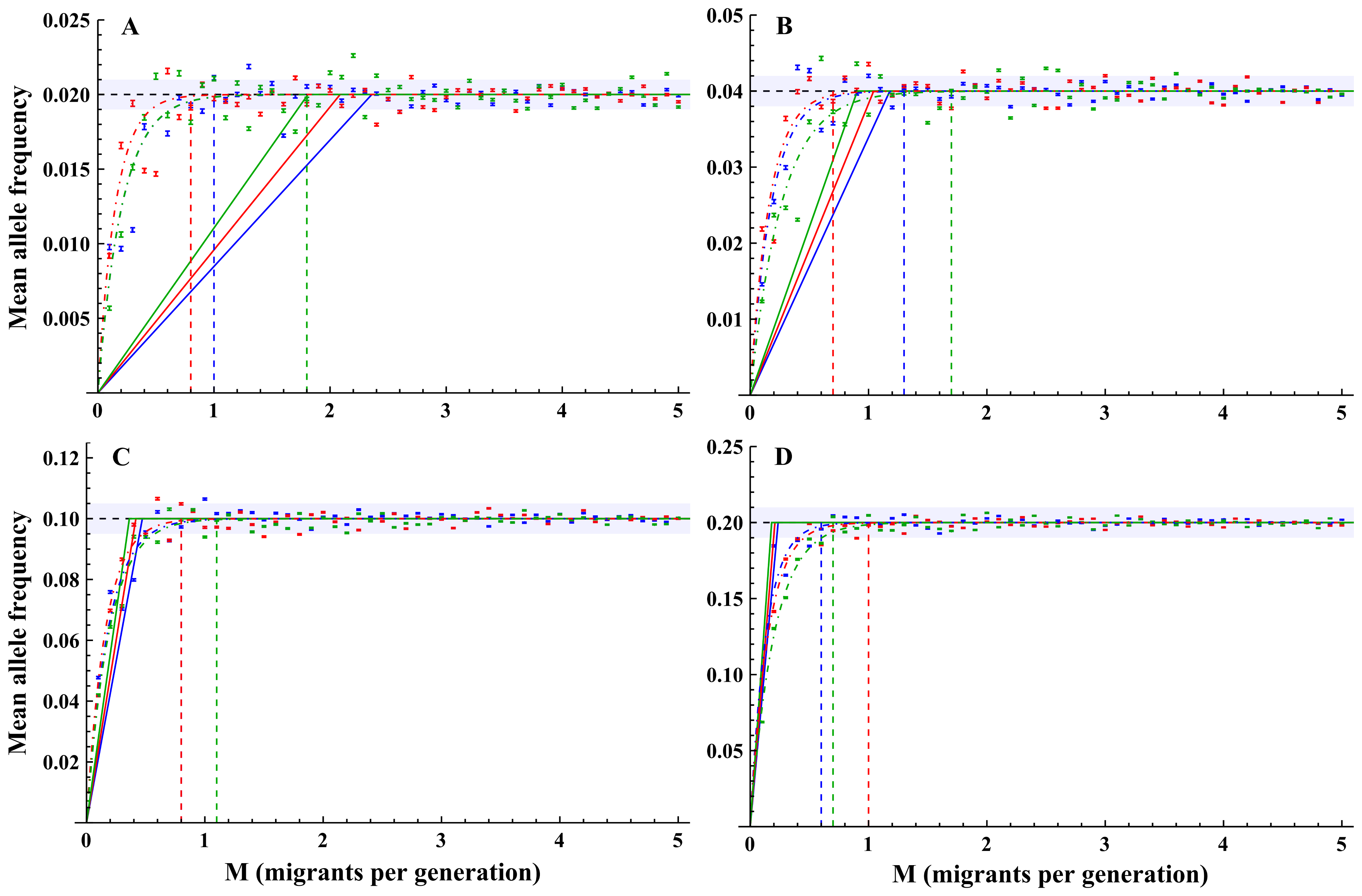

Supplement: S7 Fig — Mean allele frequencies at equilibrium () as a function of the number of migrants per generation (M). Solid lines indicate the estimation of the mean allele frequency (equation 4). Dashed lines indicate thresholds. Dashed-dotted lines indicate regression analysis results for the model ; details in S3 Table. in blue; in red; in blue. A) ; B) ; C) ; D) . Scenario parameters: . Error bars indicate the standard error of the mean. (TIFF) [file pone.0115203.s010.tiff]

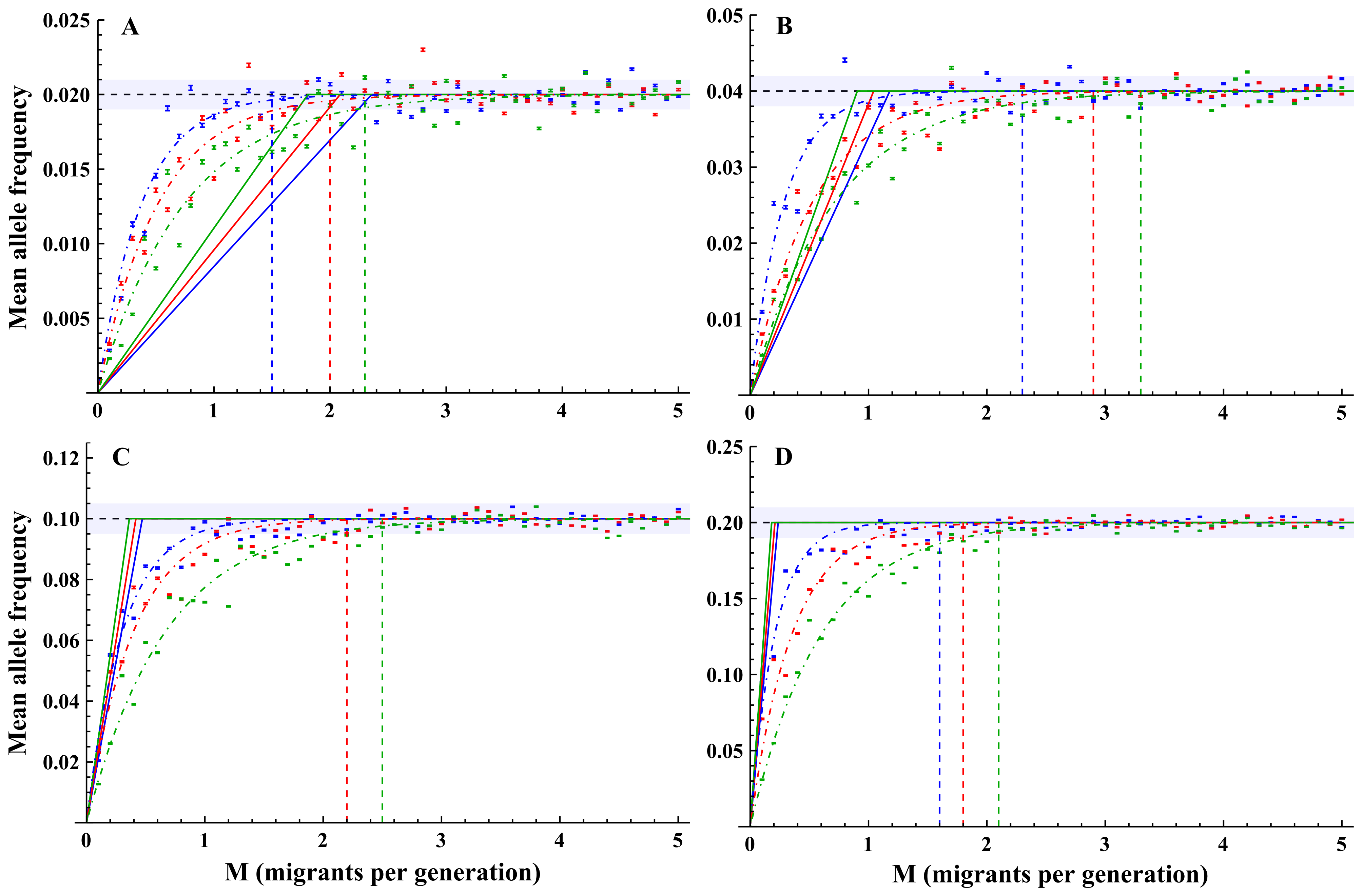

Supplement: S8 Fig — Mean allele frequencies at equilibrium () as a function of the number of migrants per generation (M). Solid lines indicate the estimation of the mean allele frequency (equation 4). Dashed lines indicate thresholds. Dashed-dotted lines indicate regression analysis results for the model ; details in S3 Table. in blue; in red; in blue. A) ; B) ; C) ; D) . Scenario parameters: . Error bars indicate the standard error of the mean. (TIFF) [file pone.0115203.s011.tiff]

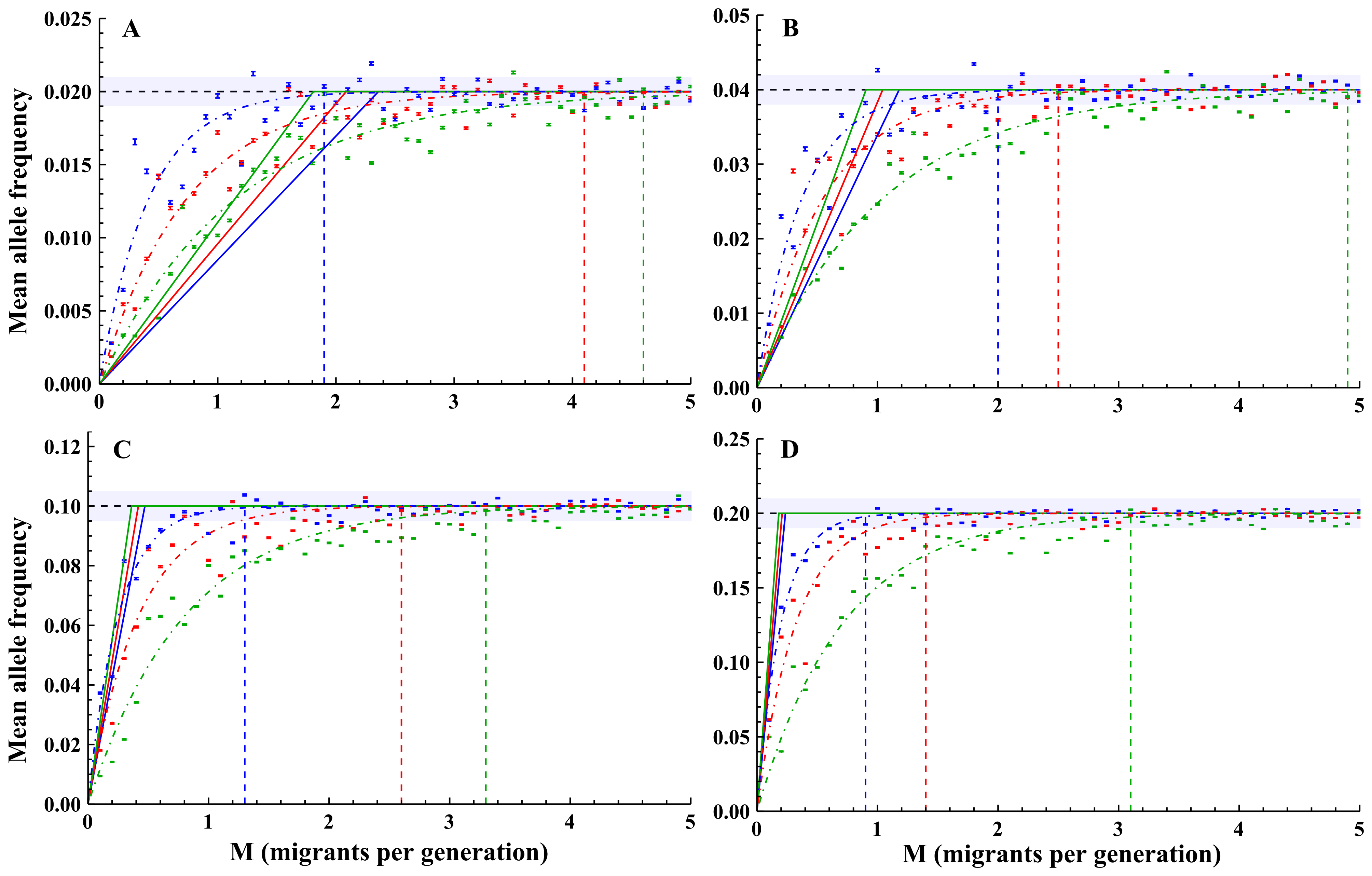

Supplement: S9 Fig — Mean allele frequencies at equilibrium () as a function of the number of migrants per generation (M). Solid lines indicate the estimation of the mean allele frequency (equation 4). Dashed lines indicate thresholds. Dashed-dotted lines indicate regression analysis results for the model ; details in S3 Table. in blue; in red; in blue. A) ; B) ; C) ; D) . Scenario parameters: . Error bars indicate the standard error of the mean. (TIFF) [file pone.0115203.s012.tiff]
