# Supplementary material for: Allelic Richness following Population Founding Events – A Stochastic Modeling Framework Incorporating Gene Flow and Genetic Drift
Source: PLoS One. 2014 Dec 19;9(12):e115203. doi: 10.1371/journal.pone.0115203 (PMC4272294; doi:10.1371/journal.pone.0115203)
Supplement: S1 Table — and thresholds for different scenarios with deterministic migration pattern. Parameters: initial population size (); growth rate (r); carrying capacity (K); source population allele frequency (Q); minimal number of migrants from source population to founded population required to reach mean allele frequency Q at equilibrium (); minimal number of migrants from source population to founded population required to reach 95% probability of presence of the allele at equilibrium (). (DOCX) [file pone.0115203.s013.docx]

Table S1. $M_{mean}$ and $M_{presence}$ thresholds for different scenarios with deterministic migration pattern

|  | | | | | $M_{mean}$ | | | | $M_{presence}$ | | | |
| --- | --- | --- | --- | --- | --- | --- | --- | --- | --- | --- | --- | --- |
| $N_{0}$ | *r* | | *K* | | *$Q=0.02$* | *$Q=0.04$* | *$Q=0.1$* | *$Q=0.2$* | *$Q=0.02$* | *$Q=0.04$* | *$Q=0.1$* | *$Q=0.2$* |
| 5 | | 0.01 | | 200 | 1.4 | 0.6 | 0.1 | 0.1 | 39.9 | 15.3 | 2.6 | 1.1 |
|  |  |  |  | 400 | 0.1 | 0.6 | 0.2 | 0.1 | 18.7 | 8.5 | 2.5 | 1.1 |
|  |  |  |  | 1000 | 0.1 | 0.8 | 0.5 | 0.1 | 22.6 | 7.9 | 1.2 | 1.1 |
|  |  | 0.05 | | 200 | 1.4 | 1.0 | 0.9 | 0.8 | 17.1 | 6.1 | 2.1 | 1.1 |
|  |  |  |  | 400 | 1.2 | 1.0 | 1.0 | 1.0 | 12.5 | 4.6 | 1.2 | 1.0 |
|  |  |  |  | 1000 | 2.0 | 1.3 | 1.0 | 1.0 | 9.0 | 4.0 | 1.1 | 1.0 |
|  |  | 0.1 | | 200 | 2.4 | 1.4 | 1.0 | 1.0 | 17.4 | 6.2 | 2.1 | 1.1 |
|  |  |  |  | 400 | 1.9 | 1.4 | 1.0 | 1.0 | 12.4 | 4.6 | 1.2 | 1.1 |
|  |  |  |  | 1000 | 1.5 | 1.4 | 1.8 | 1.1 | 8.8 | 4.1 | 1.2 | 1.0 |
| 10 | | 0.01 | | 200 | 0.2 | 1.5 | 0.1 | 0.1 | 20.8 | 7.7 | 2.1 | 1.1 |
|  |  |  |  | 400 | 0.7 | 0.6 | 0.7 | 0.5 | 17.9 | 5.9 | 2.1 | 1.1 |
|  |  |  |  | 1000 | 0.1 | 0.8 | 0.1 | 0.1 | 15.0 | 4.8 | 1.2 | 1.1 |
|  |  | 0.05 | | 200 | 1.7 | 1.8 | 1.0 | 1.0 | 17.2 | 6.2 | 2.1 | 1.1 |
|  |  |  |  | 400 | 2.2 | 1.7 | 1.0 | 1.3 | 12.4 | 4.8 | 1.2 | 1.0 |
|  |  |  |  | 1000 | 2.9 | 2.3 | 1.2 | 1.1 | 9.0 | 4.1 | 1.2 | 1.0 |
|  |  | 0.1 | | 200 | 3.4 | 1.9 | 2.0 | 0.9 | 17.4 | 6.4 | 2.1 | 1.1 |
|  |  |  |  | 400 | 2.3 | 3.0 | 2.0 | 2.1 | 12.6 | 4.7 | 1.2 | 1.1 |
|  |  |  |  | 1000 | 2.8 | 3.0 | 2.5 | 2.7 | 9.1 | 4.0 | 1.2 | 1.0 |
| 20 | | 0.01 | | 200 | 1.8 | 2.2 | 1.0 | 1.0 | 20.8 | 6.5 | 2.1 | 1.1 |
|  |  |  |  | 400 | 0.9 | 1.3 | 1.7 | 1.0 | 16.7 | 7.3 | 2.1 | 1.0 |
|  |  |  |  | 1000 | 1.0 | 1.4 | 1.0 | 0.9 | 9.9 | 4.1 | 1.6 | 1.0 |
|  |  | 0.05 | | 200 | 1.4 | 1.3 | 1.6 | 1.1 | 17. | 6.1 | 2.1 | 1.1 |
|  |  |  |  | 400 | 2.7 | 2.5 | 1.7 | 1.1 | 12.6 | 4.6 | 1.2 | 1.0 |
|  |  |  |  | 1000 | 2.7 | 3.0 | 2.9 | 2.6 | 9.0 | 4.0 | 1.2 | 1.0 |
|  |  | 0.1 | | 200 | 2.0 | 1.9 | 1.7 | 1.1 | 17.4 | 6.3 | 2.1 | 1.1 |
|  |  |  |  | 400 | 2.1 | 2.9 | 2.0 | 2.2 | 12.3 | 4.6 | 1.2 | 1.0 |
|  |  |  |  | 1000 | 4.4 | 3.1 | 3.8 | 3.2 | 9.0 | 4.1 | 1.2 | 1.0 |

Parameters: Initial population size ($N_{0}$); Growth rate (*r*); Carrying capacity (*K*); Source population allele frequency (*Q*); Minimal number of migrants from source population to founded population required to reach mean allele frequency *Q* at equilibrium ($M_{mean}$); Minimal number of migrants from source population to founded population required to reach 95% probability of presence of the allele at equilibrium ($M_{presence}$)
