# Supplementary material for: Allelic Richness following Population Founding Events – A Stochastic Modeling Framework Incorporating Gene Flow and Genetic Drift
Source: PLoS One. 2014 Dec 19;9(12):e115203. doi: 10.1371/journal.pone.0115203 (PMC4272294; doi:10.1371/journal.pone.0115203)
Supplement: S2 Table — Proportion of allelic richness recovered by gene flow and cut-off frequencies (Qc) for deterministic migration pattern. Initial population size (); growth rate (r); carrying capacity (K); migrants per generation from source population to founded population (M); cut-off frequency (Qc); source population allele frequency spectrum (defined by equation 6) with given θ value. (DOCX) [file pone.0115203.s014.docx]

Table S2. Proportion of allelic richness recovered by gene flow and cut-off frequencies (*Q_c_*) for deterministic migration pattern

|  |  |  | ***$M=1$*** | | | | ***$M=5$*** | | | | ***$M=20$*** | | | |
| --- | --- | --- | --- | --- | --- | --- | --- | --- | --- | --- | --- | --- | --- | --- |
|  |  |  |  | Proportion of allelic richness recovered | | |  | Proportion of allelic richness recovered | | |  | Proportion of allelic richness recovered | | |
| $N_{0}$ | *r* | *K* | *Q_c_* | $\theta=0.1$ | $\theta=1$ | $\theta=10$ | *Q_c_* | $\theta=0.1$ | $\theta=1$ | $\theta=10$ | *Q_c_* | $\theta=0.1$ | $\theta=1$ | $\theta=10$ |
| 5 | 0.01 | 200 | 0.21 | 0.59 | 0.17 | 0.01 | 0.05 | 0.67 | 0.33 | 0.09 | 0.04 | 0.68 | 0.35 | 0.11 |
|  |  | 400 | 0.18 | 0.6 | 0.19 | 0.01 | 0.05 | 0.67 | 0.33 | 0.09 | 0.04 | 0.68 | 0.35 | 0.11 |
|  |  | 1000 | 0.14 | 0.61 | 0.21 | 0.02 | 0.04 | 0.68 | 0.35 | 0.11 | 0.03 | 0.7 | 0.38 | 0.15 |
|  | 0.05 | 200 | 0.16 | 0.6 | 0.2 | 0.01 | 0.05 | 0.67 | 0.33 | 0.09 | 0.02 | 0.72 | 0.42 | 0.2 |
|  |  | 400 | 0.13 | 0.62 | 0.22 | 0.02 | 0.04 | 0.68 | 0.35 | 0.11 | 0.02 | 0.72 | 0.42 | 0.2 |
|  |  | 1000 | 0.12 | 0.62 | 0.23 | 0.02 | 0.04 | 0.68 | 0.35 | 0.11 | 0.02 | 0.72 | 0.42 | 0.2 |
|  | 0.1 | 200 | 0.16 | 0.6 | 0.2 | 0.01 | 0.05 | 0.67 | 0.33 | 0.09 | 0.02 | 0.72 | 0.42 | 0.2 |
|  |  | 400 | 0.14 | 0.61 | 0.21 | 0.02 | 0.04 | 0.68 | 0.35 | 0.11 | 0.02 | 0.72 | 0.42 | 0.2 |
|  |  | 1000 | 0.12 | 0.62 | 0.23 | 0.02 | 0.04 | 0.68 | 0.35 | 0.11 | 0.02 | 0.72 | 0.42 | 0.2 |
| 10 | 0.01 | 200 | 0.17 | 0.6 | 0.19 | 0.01 | 0.07 | 0.65 | 0.29 | 0.06 | 0.03 | 0.7 | 0.38 | 0.15 |
|  |  | 400 | 0.16 | 0.6 | 0.2 | 0.01 | 0.06 | 0.66 | 0.31 | 0.07 | 0.03 | 0.7 | 0.38 | 0.15 |
|  |  | 1000 | 0.17 | 0.6 | 0.19 | 0.01 | 0.05 | 0.67 | 0.33 | 0.09 | 0.03 | 0.7 | 0.38 | 0.15 |
|  | 0.05 | 200 | 0.15 | 0.61 | 0.21 | 0.01 | 0.05 | 0.67 | 0.33 | 0.09 | 0.02 | 0.72 | 0.42 | 0.2 |
|  |  | 400 | 0.14 | 0.61 | 0.21 | 0.02 | 0.04 | 0.68 | 0.35 | 0.11 | 0.02 | 0.72 | 0.42 | 0.2 |
|  |  | 1000 | 0.12 | 0.62 | 0.23 | 0.02 | 0.04 | 0.68 | 0.35 | 0.11 | 0.02 | 0.72 | 0.42 | 0.2 |
|  | 0.1 | 200 | 0.16 | 0.6 | 0.2 | 0.01 | 0.05 | 0.67 | 0.33 | 0.09 | 0.02 | 0.72 | 0.42 | 0.2 |
|  |  | 400 | 0.14 | 0.61 | 0.21 | 0.02 | 0.04 | 0.68 | 0.35 | 0.11 | 0.02 | 0.72 | 0.42 | 0.2 |
|  |  | 1000 | 0.13 | 0.62 | 0.22 | 0.02 | 0.04 | 0.68 | 0.35 | 0.11 | 0.02 | 0.72 | 0.42 | 0.2 |
| 20 | 0.01 | 200 | 0.17 | 0.6 | 0.19 | 0.01 | 0.06 | 0.66 | 0.31 | 0.07 | 0.03 | 0.7 | 0.38 | 0.15 |
|  |  | 400 | 0.16 | 0.6 | 0.2 | 0.01 | 0.06 | 0.66 | 0.31 | 0.07 | 0.02 | 0.72 | 0.42 | 0.2 |
|  |  | 1000 | 0.16 | 0.6 | 0.2 | 0.01 | 0.06 | 0.66 | 0.31 | 0.07 | 0.02 | 0.72 | 0.42 | 0.2 |
|  | 0.05 | 200 | 0.15 | 0.61 | 0.21 | 0.01 | 0.05 | 0.67 | 0.33 | 0.09 | 0.02 | 0.72 | 0.42 | 0.2 |
|  |  | 400 | 0.14 | 0.61 | 0.21 | 0.02 | 0.04 | 0.68 | 0.35 | 0.11 | 0.02 | 0.72 | 0.42 | 0.2 |
|  |  | 1000 | 0.13 | 0.62 | 0.22 | 0.02 | 0.04 | 0.68 | 0.35 | 0.11 | 0.02 | 0.72 | 0.42 | 0.2 |
|  | 0.1 | 200 | 0.16 | 0.6 | 0.2 | 0.01 | 0.05 | 0.67 | 0.33 | 0.09 | 0.02 | 0.72 | 0.42 | 0.2 |
|  |  | 400 | 0.15 | 0.61 | 0.21 | 0.01 | 0.04 | 0.68 | 0.35 | 0.11 | 0.02 | 0.72 | 0.42 | 0.2 |
|  |  | 1000 | 0.13 | 0.62 | 0.22 | 0.02 | 0.04 | 0.68 | 0.35 | 0.11 | 0.02 | 0.72 | 0.42 | 0.2 |

Initial population size ($N_{0}$); Growth rate (*r*); Carrying capacity (*K*); Migrants per generation from source population to founded population (*M*); Cut-off frequency (*Q_c_*); Source population allele frequency spectrum (defined by equation 6) with given *θ* value
