# Supplementary material for: Allelic Richness following Population Founding Events – A Stochastic Modeling Framework Incorporating Gene Flow and Genetic Drift
Source: PLoS One. 2014 Dec 19;9(12):e115203. doi: 10.1371/journal.pone.0115203 (PMC4272294; doi:10.1371/journal.pone.0115203)
Supplement: S3 Table — Fitted regression models for for mean allele frequency at equilibrium. Curves shown in S1-S9 Figs. (DOCX) [file pone.0115203.s015.docx]

|  |  |  | $Q=0.02$ | | $Q=0.04$ | | $Q=0.1$ | | $Q=0.2$ | |
| --- | --- | --- | --- | --- | --- | --- | --- | --- | --- | --- |
| $N_{0}$ | *r* | *K* | α | $R^{2}$ | α | $R^{2}$ | α | $R^{2}$ | α | $R^{2}$ |
| 5 | 0.01 | 200 | 28.67 | 0.997 | 25.48 | 0.999 | 22.77 | 1. | 28.34 | 1. |
|  |  | 400 | 13.59 | 0.998 | 18.66 | 0.998 | 17.29 | 0.999 | 22.59 | 1. |
|  |  | 1000 | 31.71 | 0.995 | 16.32 | 0.998 | 14.46 | 0.999 | 16.8 | 1. |
|  | 0.05 | 200 | 4.84 | 0.997 | 5.42 | 0.998 | 5.59 | 0.999 | 9.21 | 0.999 |
|  |  | 400 | 5.12 | 0.994 | 4.62 | 0.997 | 4.66 | 0.999 | 5.75 | 1. |
|  |  | 1000 | 4.34 | 0.996 | 4.1 | 0.997 | 4.1 | 0.999 | 4.14 | 0.999 |
|  | 0.1 | 200 | 3.59 | 0.997 | 3.28 | 0.999 | 3.94 | 0.999 | 4.4 | 0.999 |
|  |  | 400 | 3.02 | 0.993 | 2.72 | 0.997 | 2.92 | 0.999 | 3.32 | 0.999 |
|  |  | 1000 | 2.61 | 0.994 | 2.25 | 0.996 | 2.37 | 0.999 | 2.52 | 0.999 |
| 10 | 0.01 | 200 | 29.85 | 0.997 | 8.92 | 0.999 | 13.09 | 1. | 12.79 | 1. |
|  |  | 400 | 9.62 | 0.997 | 11.43 | 0.999 | 11.51 | 0.999 | 9.52 | 1. |
|  |  | 1000 | 5.49 | 0.996 | 10.61 | 0.998 | 10.26 | 0.999 | 10.95 | 1. |
|  | 0.05 | 200 | 3.51 | 0.997 | 3.94 | 0.998 | 4.82 | 0.998 | 4.65 | 0.999 |
|  |  | 400 | 3.09 | 0.995 | 2.71 | 0.998 | 2.99 | 0.999 | 3.08 | 0.999 |
|  |  | 1000 | 2.36 | 0.995 | 2.49 | 0.998 | 2.26 | 0.999 | 2.4 | 0.999 |
|  | 0.1 | 200 | 2.75 | 0.996 | 2.87 | 0.996 | 4.17 | 0.999 | 5.08 | 0.999 |
|  |  | 400 | 1.84 | 0.996 | 1.96 | 0.995 | 2.19 | 0.998 | 2.76 | 0.998 |
|  |  | 1000 | 1.5 | 0.994 | 1.33 | 0.998 | 1.49 | 0.999 | 1.65 | 0.998 |
| 20 | 0.01 | 200 | 4.75 | 0.994 | 5.56 | 0.998 | 5.54 | 0.999 | 7.47 | 0.999 |
|  |  | 400 | 6.52 | 0.996 | 6.22 | 0.998 | 6.29 | 0.999 | 6.56 | 1. |
|  |  | 1000 | 4.77 | 0.996 | 3.76 | 0.996 | 5.2 | 0.999 | 4.95 | 1. |
|  | 0.05 | 200 | 2.67 | 0.997 | 3.51 | 0.998 | 3.26 | 0.999 | 4.83 | 0.999 |
|  |  | 400 | 1.94 | 0.996 | 1.92 | 0.997 | 2.5 | 0.998 | 2.84 | 0.999 |
|  |  | 1000 | 1.35 | 0.995 | 1.42 | 0.997 | 1.49 | 0.998 | 1.67 | 0.999 |
|  | 0.1 | 200 | 2.41 | 0.993 | 2.71 | 0.996 | 3.99 | 0.998 | 4.99 | 0.999 |
|  |  | 400 | 1.36 | 0.995 | 1.84 | 0.994 | 2.38 | 0.996 | 3.09 | 0.997 |
|  |  | 1000 | 0.84 | 0.996 | 0.95 | 0.996 | 1.27 | 0.996 | 1.49 | 0.998 |

Table S3 – Fitted regression models for $Q(1-e^{-\alpha M})$ for mean allele frequency at equilibrium for the scenarios simulated. Curves shown in figures S1-S9.
